# A Krüppel-like factor is required for development and regeneration of germline and yolk cells from somatic stem cells in planarians

**DOI:** 10.1371/journal.pbio.3001472

**Published:** 2022-07-15

**Authors:** Melanie Issigonis, Akshada B. Redkar, Tania Rozario, Umair W. Khan, Rosa Mejia-Sanchez, Sylvain W. Lapan, Peter W. Reddien, Phillip A. Newmark

**Affiliations:** 1 Morgridge Institute for Research, Madison, Wisconsin, United States of America; 2 Program in Cellular and Molecular Biology, University of Wisconsin-Madison, Madison, Wisconsin, United States of America; 3 Department of Integrative Biology, University of Wisconsin-Madison, Madison, Wisconsin, United States of America; 4 Whitehead Institute for Biomedical Research, Cambridge, Massachusetts, United States of America; 5 Department of Biology, Massachusetts Institute of Technology, Cambridge, Massachusetts, United States of America; 6 Howard Hughes Medical Institute, Massachusetts Institute of Technology, Cambridge, Massachusetts, United States of America; 7 Howard Hughes Medical Institute, University of Wisconsin-Madison, Madison, Wisconsin, United States of America; Institute of Molecular Biology, GERMANY

## Abstract

Sexually reproducing animals segregate their germline from their soma. In addition to gamete-producing gonads, planarian and parasitic flatworm reproduction relies on yolk cell–generating accessory reproductive organs (vitellaria) supporting development of yolkless oocytes. Despite the importance of vitellaria for flatworm reproduction (and parasite transmission), little is known about this unique evolutionary innovation. Here, we examine reproductive system development in the planarian *Schmidtea mediterranea*, in which pluripotent stem cells generate both somatic and germ cell lineages. We show that a homolog of the pluripotency factor Klf4 is expressed in primordial germ cells (PGCs), presumptive germline stem cells (GSCs), and yolk cell progenitors. Knockdown of this *klf4-like* (*klf4l*) gene results in animals that fail to specify or maintain germ cells; surprisingly, they also fail to maintain yolk cells. We find that yolk cells display germ cell–like attributes and that vitellaria are structurally analogous to gonads. In addition to identifying a new proliferative cell population in planarians (yolk cell progenitors) and defining its niche, our work provides evidence supporting the hypothesis that flatworm germ cells and yolk cells share a common evolutionary origin.

## Introduction

Sexually reproducing animals consist of 2 main cell types: germ cells that produce gametes (eggs and sperm) and somatic cells that make up the remainder of the body. Animal germ cells are typically specified in either of 2 ways: by determinate or inductive specification [1–4]. Determinate specification results from the segregation of specialized maternal determinants (germ plasm) at the onset of embryogenesis; those cells receiving germ plasm acquire germ cell fate. In contrast, inductive specification occurs later in embryogenesis when extrinsic signals from surrounding tissues instruct competent cells to form germ cells. Determinate specification has been studied extensively in traditional laboratory models, including *Drosophila*, *Caenorhabditis elegans*, zebrafish, and frogs [5–17]. Inductive specification, although intensively investigated in mammals, has been less well characterized mechanistically across phylogeny, even though it is the basal and most common mode of germ cell specification in the animal kingdom [1–3].

Irrespective of the mode of germ cell specification, an important commonality exists: Once formed, germ cells are set aside from the soma. The developmental decision to segregate the germ cell lineage from somatic cells is essential for species continuity; unlike the soma, which expires each generation, “immortal” germ cells pass on genetic information and serve as a perpetual link between generations. Many animals (e.g., *Drosophila*, *C*. *elegans*, and mice) specify their germ cells (and segregate them from their soma) only once during embryonic development [1–4]. However, some animals retain the ability to specify new germ cells throughout their lifetime. Sponges and cnidarians maintain into adulthood multipotent stem cells that fuel the continuous production of new germ cells while also giving rise to somatic cell lineages [18–24]. How do these stem cells decide between somatic and germ cell fates?

Planarian flatworms can regenerate an entire body from small tissue fragments. Intensive efforts have been devoted to understanding the mechanisms underlying this regenerative prowess. Planarian regeneration is driven by pluripotent stem cells called neoblasts that are distributed throughout the body [25–27]. Planarians can also inform our understanding of germ cell biology: The neoblasts that give rise to all somatic lineages also give rise to new germ cells [28–31]. Interestingly, neoblasts and germ cells express a shared set of conserved “germline genes,” including *piwi*, *vasa*, *pumilio*, and *tudor* [32,33], which play important roles in neoblast function [34–44]. Like mammals, planarians undergo inductive germ cell specification [28–31,45,46]. However, the mechanistic basis underlying germ cell specification from “somatic” neoblasts and the factors involved in adopting somatic versus germ cell fate remain obscure.

Here, we investigate how new germ cells are specified from neoblasts throughout postembryonic development and during regeneration in planarians. We also examine another critical aspect of the planarian reproductive system: the development of an extensive network of accessory organs known as vitellaria. Unique among animals, eggs of most flatworms are ectolecithal: Yolk is not present within oocytes themselves, but rather is made by vitellaria that produce specialized yolk cells (vitelline cells or vitellocytes). Planarians and all parasitic flatworms are characterized by ectolecithality. However, despite the importance of vitellaria in the life cycle and transmission of these parasites [47,48], little is known about the development of vitellaria or production of yolk cells.

We show that a homolog of the conserved transcription factor *Krüppel-like factor 4* (*klf4*), a critical inducer of pluripotency in mammals [49], is expressed in male and female presumptive germline stem cells (GSCs) in the planarian *Schmidtea mediterranea*, as well as in a newly discovered population of mitotically competent yolk cell progenitors. We demonstrate that *klf4-like* (*klf4l*) is required for germ cell specification and that *klf4l* knockdown leads to the loss of both germ cell and yolk cell lineages. We provide evidence that yolk cell–producing organs in planarians consist of 2 distinct cell types: a yolk cell lineage, which is characterized by several germ cell–like attributes, and support cells, which sustain yolk cell maintenance and differentiation. Our data show that planarian vitellaria are structurally analogous to gonads and that yolk cells share several important features with both somatic neoblasts and germ cells.

## Results

### *klf4l* is expressed in planarian gonads and yolk-producing accessory organs

**Fig 1:**
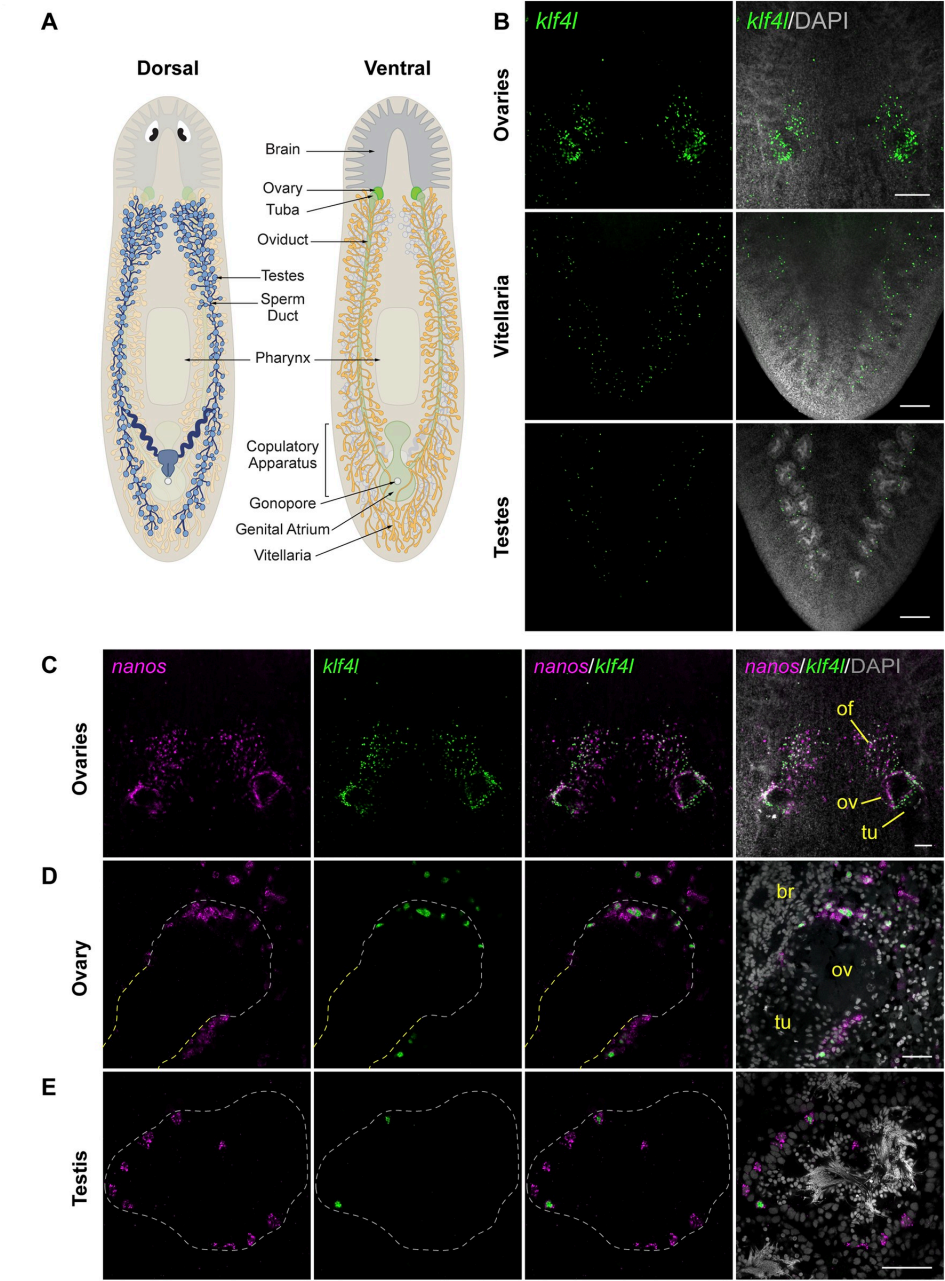
*klf4l* is expressed in gonads and vitellaria and is restricted to a subset of *nanos*^*+*^ germ cells in planarian ovaries and testes. (**A**) Schematics depicting the dorsal (left) and ventral (right) views of landmark structures and various reproductive organs in adult sexual *S*. *mediterranea*. (**B**) Maximum intensity projections of confocal sections showing FISH of *klf4l* (green) in ventral head region (top), ventral tail region (middle), and dorsal tail region (bottom). (**C**) Maximum intensity projection of confocal sections showing dFISH of *klf4l* (green) and germline marker *nanos* (magenta) in ventral head region. *klf4l-* and *nanos*-expressing cells are detected surrounding the tuba (tu) at the base of each ovary (ov), along the periphery of the ovaries, and in anterior ovarian fields (of) situated mediolaterally along the brain. (**D**) Single confocal section of a planarian ovary located posterior to the brain (br) showing *klf4l* (green) and *nanos* (magenta) dFISH. *klf4l-* and *nanos*-expressing cells are found at the ovary-tuba junction, along the periphery of the ovary, and in germ cells anterior to the ovary. Dashed line denotes ovary (white) and tuba (yellow) boundary. (**E**) Confocal section of *klf4l* (green) and *nanos* (magenta) dFISH showing *klf4l*/*nanos* double-positive and *nanos* single-positive cells along the periphery of the testis. Dashed line denotes testis boundary. (**B–E**) Nuclei are counterstained with DAPI (gray). Scale bars, 200 μm (**B**), 100 μm (**C**), and 50 μm (**D**, **E**). dFISH, double FISH; FISH, fluorescent RNA in situ hybridization; *klf4l*, *klf4-like*.

In the search for regulators of germ cell fate in planarians, we focused on the conserved transcription factor KLF4, a key pluripotency factor in mammals [49]. Sexual *S*. *mediterranea* are cross-fertilizing, simultaneous hermaphrodites. Using fluorescent RNA in situ hybridization (FISH) on sexually mature adults, we found that one of the 5 *klf* genes present in the *S*. *mediterranea* genome, *klf4l* (S1A and S1B Fig), is expressed at high levels within the ventrally situated ovaries, as well as in cells that are distributed along the medial posterior region of each lobe of the cephalic ganglia and appear to be arranged in a field anterior to each ovary (Fig 1A and 1B). Sparse *klf4l*^*+*^ cells are also located dorsolaterally, where the testes reside (Fig 1A and 1B). Additionally, *klf4l*^*+*^ cells are scattered ventrolaterally throughout the parenchyma (the tissue surrounding the planarian’s internal organs), in a pattern reminiscent of vitellaria, the yolk-producing organs essential for reproduction (Fig 1A and 1B). Thus, this pluripotency-associated transcription factor is expressed in areas associated with male and female reproductive tissues.

### *klf4l* expression is restricted to a subset of *nanos*^*+*^ germ cells in ovaries and testes

To analyze gonadal *klf4l* expression in more detail, we performed double FISH (dFISH) to detect *klf4l* and the germline marker *nanos* [30] (Fig 1C–1E). Previous work in planarians has shown that gonadal *nanos* expression is restricted to the early spermatogonia and oogonia in the outermost layer of testes and ovaries, respectively, which have been interpreted as presumptive GSCs [29,46,50,51]. In addition to the previously described *nanos*^*+*^ germ cells found at the ovary periphery, we detected *nanos*^*+*^ cells in the same anterior ovarian fields described above (Fig 1C); a substantial proportion of *nanos*^*+*^ cells in these fields coexpresses *klf4l* (81%, *n* = 1,116 *nanos*^*+*^ cells) and all *klf4l*^*+*^ cells are *nanos*^*+*^ (100%, *n* = 908 *klf4l*^*+*^ cells) (S1 Data). *klf4l* expression is similarly restricted to a subset of *nanos*^*+*^ germ cells located at the ovary periphery, and in *nanos*^*+*^ cells clustered at the boundary between the ovary and its outlet, the tuba (the anterior-most portion of the oviduct where fertilization occurs) (Fig 1D) (90%, *n* = 1,588 *nanos*^*+*^ cells). All *klf4l*^*+*^ cells within and at the base of the ovary coexpress *nanos* (100%, *n* = 1,423 *klf4l*^*+*^ cells) (S1 Data). In the testes of sexually mature adults, *klf4l* is also expressed around the periphery, but is confined to a small subset of *nanos*^*+*^ germ cells (14%, *n* = 10,628 *nanos*^*+*^ cells) (Fig 1E). Similar to female germ cells, all *klf4l*^*+*^ male germ cells express *nanos* (100%, *n* = 1,475 *klf4l*^*+*^ cells) (S1 Data). Our observations show that in both ovaries and testes, the *nanos*^*+*^ presumptive GSCs are heterogeneous with respect to *klf4l* expression.

Since only a fraction of *nanos*^*+*^ germ cells express *klf4l*, we wondered whether *klf4l* expression represents the earliest stages of *nanos*^*+*^ germ cell development. To answer this question, we examined the developmental progression of *klf4l* and *nanos* expression, starting from the emergence of primordial germ cells (PGCs) in newly hatched planarians. Previous studies describing *nanos* expression in hatchlings failed to detect the presence of female (i.e., anteroventral) PGCs in planarians until 1 week posthatching. Male (dorsolateral) *nanos*^*+*^ PGCs were observed in a minority of planarians during the final stages of embryonic development (stage 8 embryos) and in 1-day-old hatchlings [30,51]. In contrast to these studies, by FISH, we were able to detect female *nanos*^*+*^ cells in 100% of 1-day-old hatchlings; however, only a fraction of these cells expresses the neoblast/germline marker *piwi-1*, (40%, *n* = 1,199 *nanos*^*+*^ cells), indicating that not all anteroventral *nanos*^*+*^ cells are germ cells (S2A Fig). While predominantly expressed in germ cells, *nanos* transcripts have also been detected in a population of eye cells [51]. Consistent with their ventral location and proximity to the cephalic ganglia, we postulate that *nanos*^*+*^/*piwi-1*^–^ cells may represent another somatic cell population, such as neurons. To determine the proportion of *nanos*^*+*^ PGCs that express *klf4l*, we performed triple FISH and found that 56% of *nanos*^*+*^/*piwi-1*^*+*^ PGCs coexpress *klf4l* (*n* = 598 *nanos*^*+*^/*piwi-1*^*+*^ cells) (S2A Fig), indicating that this heterogeneity persists throughout sexual development (72% and 75% of *nanos*^*+*^/*piwi-1*^*+*^ germ cells are *klf4l*^*+*^ in immature, juvenile ovaries and mature, adult ovaries, respectively) (S2B Fig).

**Fig 2:**
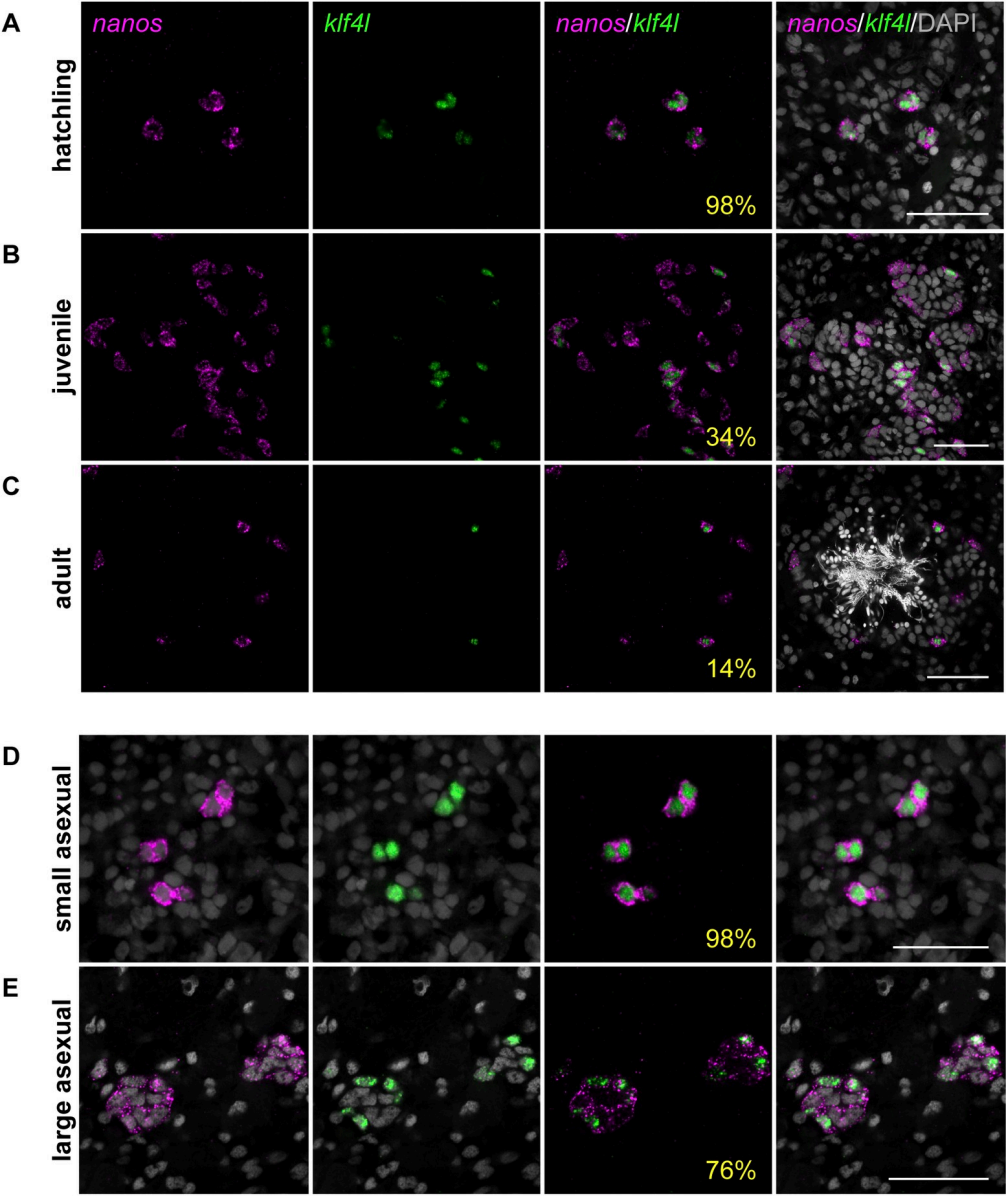
*klf4l* expression becomes restricted to a subset of *nanos*^*+*^ male germ cells during sexual planarian maturation and asexual planarian growth. (**A–C**) Confocal sections showing dFISH of *klf4l* (green) and *nanos* (magenta) in hatchling testis primordia (**A**), juvenile testes (**B**), and a sexually mature adult testis (**C**). *klf4l* is expressed in most *nanos*^*+*^ male PGCs in hatchlings and becomes progressively restricted to a subpopulation of *nanos*^*+*^ germ cells as planarians sexually mature. (**D**, **E**) Confocal sections showing dFISH of *klf4l* (green) and *nanos* (magenta) in testis primordia in small (**D**) and large (**E**) asexual planarians. *klf4l* is coexpressed in almost all *nanos*^*+*^ male germ cells in small asexuals and is restricted to a subset of *nanos*^*+*^ male germ cells in large asexuals. (**A–E**) Percentages reflect *nanos*^*+*^ cells that are also *klf4l*^*+*^. Nuclei are counterstained with DAPI (gray). Scale bars, 50 μm. Underlying data can be found in S1 Data. dFISH, double FISH; *klf4l*, *klf4-like*; PGC, primordial germ cell.

Male germ cells are easily observed throughout testis maturation. In hatchlings, all *nanos*^*+*^ cells distributed dorsolaterally (where testes will develop) coexpress *piwi-1* (100%, *n* = 517 *nanos*^*+*^ PGCs) (S1 Data). Essentially all *nanos*^*+*^ PGCs also coexpress *klf4l* (98%, *n* = 189 *nanos*^*+*^ cells) (Fig 2A). However, as planarians undergo sexual maturation and testis primordia continue to develop, *klf4l* is expressed in an increasingly smaller proportion of the *nanos*^+^ population (34%, *n* = 5,198 *nanos*^*+*^ cells in juveniles and 14%, *n* = 10,628 *nanos*^*+*^ cells in adults), and there is a marked increase in *klf4l*^–^/*nanos*^*+*^ germ cells (Fig 2B and 2C). These data indicate that in hatchlings, newly specified PGCs express both *klf4l* and *nanos*; whereas, during sexual development a *nanos* single-positive germ cell population emerges and expands. These observations are consistent with a model in which *klf4l*^*+*^/*nanos*^*+*^ cells represent the most undifferentiated germ cell state (i.e. PGC and GSC), and *klf4l*^–^/*nanos*^*+*^ germ cells are their immediate progeny. It is important to note that essentially all *klf4l*^*+*^ cells coexpressed *nanos* and *piwi-1* (S1 Data)—we did not detect a population of neoblasts (*nanos*^–^/*piwi-1*^*+*^) that expressed *klf4l*.

Thus far, we have characterized *klf4l* expression in the sexual strain of *S*. *mediterranea*. However, this species also exists as an obligate asexual biotype, which reproduces exclusively by fission and does not produce mature gametes or accessory reproductive organs. Although asexual planarians do not develop functional gametes, they nevertheless specify PGCs in small clusters of *nanos*^*+*^ gonadal primordia. These *nanos*^*+*^ cells do not proliferate or differentiate [29,30,51]. By comparing small (approximately 2 mm) and large (>5 mm) asexuals, we found that the number of *nanos*^*+*^ germ cells in female (anteroventrally located) and male (dorsolaterally located) primordia increases as animals grow (Figs 2D, 2E, S2C and S2D). We examined whether *klf4l* expression was restricted to these early PGCs, and by dFISH we found that *klf4l* is coexpressed in the majority of female *nanos*^*+*^ cells, in similar proportions for both small and large asexuals (91%, *n* = 24 *nanos*^*+*^ cells and 86%, *n* = 213 *nanos*^*+*^ cells, respectively) (S2C and S2D Fig). In contrast, *klf4l* is coexpressed in virtually all male *nanos*^*+*^ cells in small asexuals (98%, *n* = 559 *nanos*^*+*^ cells), whereas testis primordia in larger animals contain both *klf4l*^*+*^/*nanos*^*+*^ cells and *klf4l*^–^/*nanos*^*+*^ cells (76% and 24% respectively, *n* = 1645 *nanos*^*+*^ cells) (Fig 2D and 2E). Thus, in both growing asexuals and maturing sexuals, as *nanos*^*+*^ cells in testis primordia increase in number, *klf4l* expression becomes restricted to a subset of these germ cells. This similarity suggests that in the asexual biotype germ cells can undergo the first step of development—from *klf4l*^*+*^/*nanos*^*+*^ to *klf4l*^–^/*nanos*^*+*^ cells—before reaching a block in differentiation.

### *klf4l*-expressing germ cells in ovaries and testes are mitotically active

In many animals, the production of gametes in adulthood is enabled by GSCs. Our findings raise the possibility that *klf4l*-expressing cells are GSCs representing the top of oogonial and spermatogonial lineages. All GSCs have the ability to undergo self-renewing divisions, which give rise to differentiating daughter cells while maintaining the stem cell pool. By combining phospho-Histone H3 (pHH3) immunostaining with *klf4l* and *nanos* dFISH, we examined the mitotic profiles of cells within the germ cell hierarchy and sought to ascertain whether *klf4l*^*+*^/*nanos*^*+*^ cells are competent to divide and, therefore, fulfill a basic criterion of GSC behavior.

**Fig 3:**
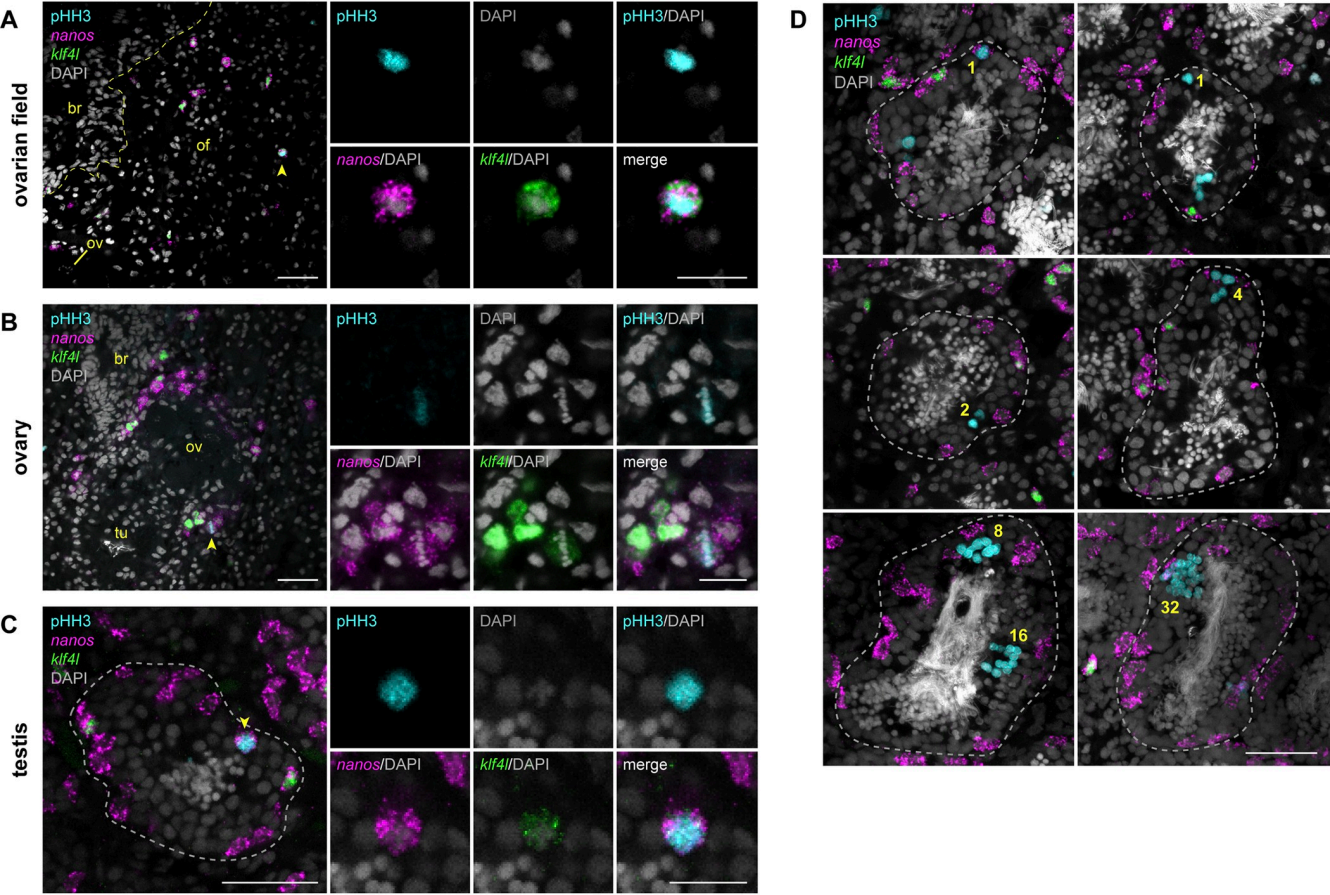
*klf4l*^*+*^ germ cells in planarian ovaries and testes are mitotically active. (**A–C**) Confocal sections showing dFISH of *klf4l* (green) and *nanos* (magenta) and immunostaining of mitotic marker pHH3 (cyan) in the ovarian field (of) located anterior to the ovary (ov) and proximal to the brain (br; boundary denoted by yellow dashed line) (**A**), the ovary, which is anterior to the tuba (tu) (**B**), and the testis (boundary denoted by gray dashed line) (**C**). Side panels are high magnification views of *klf4l*/*nanos*/pHH3 triple-positive cells (yellow arrowheads). (**D**) Confocal sections (top 4 panels) and maximum intensity projections (bottom 2 panels) showing dFISH of *klf4l* (green) and *nanos* (magenta) and immunostaining of pHH3 (cyan) in testes (boundary denoted by gray dashed line). Yellow numbers denote pHH3^+^ germ cells dividing throughout spermatogenesis: single *nanos*^*+*^ cell; single *nanos*^*−*^ cell; 2-, 4-, 8-cell spermatogonial cysts; and 16- and 32-cell spermatocyte cysts. (**A–D**) Nuclei are counterstained with DAPI (gray). Scale bars, 50 μm for whole-gonad images, 20 μm for side panels. *klf4l*, *klf4-like*; pHH3, phospho-Histone H3.

We found that *klf4l*^*+*^/*nanos*^*+*^ germ cells within the ovarian fields and the outer periphery of the ovaries are mitotically active (0.3%, *n* = 3409 *klf4l*^*+*^/*nanos*^*+*^ cells) (Fig 3A and 3B). We also detected proliferation of *klf4l*^–^/*nanos*^*+*^ oogonia in the ovaries, whereas *nanos*^*−*^ oogonia within the ovaries do not divide mitotically. Thus, female germ cells are specified and proliferate within the ovarian field and/or the ovary periphery, and as oogonia turn off *nanos* expression, they cease to divide mitotically and differentiate into oocytes.

Male germ cells actively divide throughout spermatogenesis; spermatogonia undergo 3 rounds of synchronous mitotic divisions with incomplete cytokinesis to produce 2-, 4-, and 8-cell spermatogonial cysts connected by intercellular bridges, whose cells differentiate into primary spermatocytes and divide meiotically to generate 32 spermatids that ultimately transform into mature sperm [31,52]. We detected pHH3^*+*^/*klf4l*^*+*^/*nanos*^*+*^ triple-positive cells in testes of both hatchlings (1%, *n* = 773 *klf4l*^*+*^/*nanos*^*+*^ cells) and adults (0.2%, *n* = 2,436 *klf4l*^*+*^/*nanos*^*+*^ cells). In mature sexuals, mitotic, single-cell spermatogonia and mitotic doublets were observed in *nanos*^*+*^ germ cells (including *klf4l*^*+*^/*nanos*^*+*^ cells) along the outermost periphery of the testis (Fig 3C and 3D). We also observed *nanos*^–^/pHH3^+^ singlets and doublets, which might represent mitotic *nanos*^*−*^ single-cells or 2-cell spermatogonia (Fig 3D). We never detected *nanos* expression in pHH3^+^ 4- or 8-cell premeiotic spermatogonial cysts, or in 16- or 32-cell meiotic cysts (Fig 3D). All our observations thus far support a model in which the spermatogonial lineage consists of *klf4l*^*+*^/*nanos*^*+*^ germ cells at the top of the hierarchy, giving rise to *klf4l*^–^/*nanos*^*+*^ and subsequently *klf4l*^–^/*nanos*^*−*^ single-cell spermatogonia and that germ cells cease expressing *nanos* once spermatogonial cystogenic divisions have occurred.

### *klf4l* is required for female and male gametogenesis and is necessary for PGC specification

**Fig 4:**
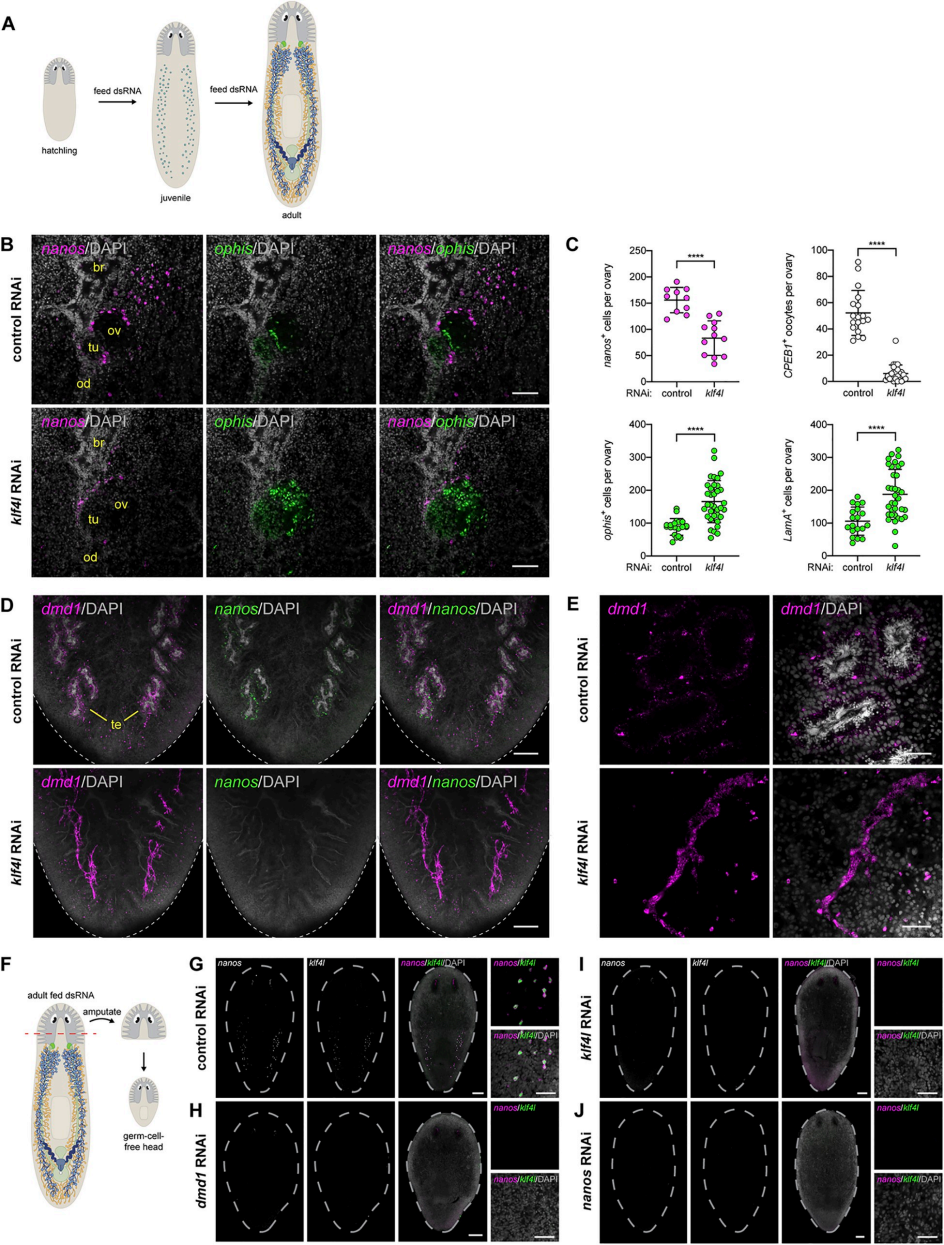
*klf4l* is required for gametogenesis in adult ovaries and testes and is necessary for PGC specification. (**A**) RNAi scheme during development in sexual *S*. *mediterranea* from newborn hatchling to sexually mature adult. (**B**) Single confocal section of an ovary (ov) located at the posterior of the brain (br) showing dFISH of *nanos* (magenta) and *ophis* (green; somatic gonadal cells, tuba (tu), oviduct (od) in control and *klf4l* RNAi planarians. (**C**) Quantification of *nanos*^*+*^ germ cells, *CPEB1*^*+*^ oocytes, *ophis*^*+*^ somatic gonadal cells, and *LamA*^*+*^ somatic gonadal cells per ovary in control and *klf4l* RNAi animals. *N* = 3 experiments. Data are presented as mean ± SD. *klf4l* RNAi results in significantly fewer germ cells and a corresponding increase in somatic support cells compared to control RNAi ovaries, *p* < 0.0001, 2-tailed Welch *t* test. Underlying data can be found in S1 Data. (**D**) Maximum intensity projections of confocal sections showing dFISH of *dmd1* (magenta; somatic gonadal cells) and *nanos* (green) in dorsal tail region. *dmd1-* and *nanos*-expressing cells are detected surrounding the DAPI-rich sperm located at the center of each testis (te) in control RNAi planarians. *dmd1*^*+*^ somatic gonadal cells are present but display a “collapsed” appearance due to the loss of germ cells in *klf4l* RNAi planarians. Dashed line denotes planarian boundary. *N* = 3 experiments, *n* = 9 to 12 planarians. (**E**) Confocal sections of control and *klf4l* RNAi testes. Note loss of spermatogenesis and “collapsed” appearance of *dmd1*^*+*^ somatic gonadal cells in *klf4l* RNAi testes compared to controls. (**F**) Amputation scheme to assay de novo respecification of germ cells. Amputation anterior to the ovaries results in a head fragment lacking any reproductive tissues (soma only). This head fragment will regenerate a new trunk and tail and will specify new germ cells. (**G–J**) Maximum intensity projections of confocal sections showing dFISH of *klf4l* (green) and *nanos* (magenta) in head regenerates 2 weeks postamputation. *N* = 3 to 5 experiments, *n* = 14 to 35 planarians. (**G**) Control RNAi head regenerates specify new *nanos*^*+*^ PGCs that coexpress *klf4l*. (**H-J**) *klf4l* and *nanos* RNAi head regenerates phenocopy *dmd1* knockdowns and fail to specify *klf4l*^*+*^/*nanos*^*+*^ PGCs. (**B, D–E, G–J**) Nuclei are counterstained with DAPI (gray). Scale bars, 100 μm (**B**), 200 μm (**D**), 50 μm (**E**), 200 μm for whole-planarian images, 50 μm for side panels (**G–J**). *klf4l*, *klf4-like*; PGC, primordial germ cell; RNAi, RNA interference.

Having established *klf4l* as an early germ cell marker, we asked whether *klf4l* is required for gonadal development. We induced *klf4l* RNA interference (RNAi) by feeding hatchlings double-stranded RNA (dsRNA) twice a week for 4 to 6 weeks—the time normally required to reach sexual maturity (Fig 4A). We examined the effects of *klf4l* knockdown on gonadal development by FISH to detect markers of early germ cells (*nanos*), oocytes (*Cytoplasmic Polyadenylation Element Binding Protein 1*, *CPEB1*), and gonadal somatic support cells (*ophis*, *Laminin A*, and *dmd1*) [50,53,54]. *klf4l* knockdown resulted in a significant reduction of early (*nanos*^*+*^) germ cells in the anterior ovarian fields and ovaries as well as a loss of mature (*CPEB1*^*+*^) oocytes (Figs 4B, 4C, and S3A). In extreme cases, ovaries were devoid of mature germ cells. Despite this dramatic loss of germ cells, *klf4l* RNAi ovaries were larger than their control RNAi counterparts, because of a significant expansion of (*ophis*^*+*^ or *LamA*^*+*^) somatic support cells (Figs 4B, 4C, and S3A). *klf4l* RNAi also led to a loss of germ cells in the testes (Fig 4D and 4E). Agametic *klf4l* RNAi testes consist of *dmd1*^*+*^ somatic cells and have a “collapsed” appearance due to the absence of germ cells (Fig 4D and 4E). To test for specificity of our RNAi phenotypes and to rule out the possibility of off-target effects, we independently targeted 2 nonoverlapping portions of *klf4l* (amino terminus versus carboxyl terminus), which induced similar levels of mRNA knockdown (S4 Fig) and indistinguishable phenotypes (S5A and S5B Fig). Taken together, these data indicate that *klf4l* is required for the maintenance of *nanos*^*+*^ germ cells to sustain proper gametogenesis in both ovaries and testes.

Is *klf4l* also necessary for PGC specification? Since *klf4l*^*+*^/*nanos*^*+*^ PGCs are specified during embryogenesis and are already present in newborn hatchlings, it is not feasible to induce RNAi by dsRNA feeding before PGC specification. However, planarians can regenerate germ cells de novo—amputated head fragments comprised solely of somatic cells can inductively specify new germ cells [28–30]. Therefore, to test the requirement of a gene during PGC specification, one can feed adult planarians dsRNA to induce RNAi, amputate heads anterior to all reproductive tissues, and examine regenerating “germ cell-free” head fragments for de novo germ cell specification (Fig 4F) [50]. Two weeks postamputation, we found that control head fragments respecified *klf4l*^*+*^*/nanos*^*+*^ cells dorsolaterally (Figs 4G and S3B) [30]. By contrast, respecification of PGCs in *klf4l* RNAi or *nanos* RNAi head fragments was significantly impaired, with several head regenerates lacking germ cells entirely (Figs 4I, 4J, and S3B). Similarly, *dmd1* RNAi head fragments fail to specify new *klf4l*^*+*^/*nanos*^*+*^ germ cells during regeneration (Figs 4H and S3B), because *dmd1* (a critical somatic gonadal niche factor) is required nonautonomously for germ cell specification [50]. These data suggest that *klf4l* and *nanos* are required cell autonomously for specification of new germ cells from neoblasts and that *klf4l*^*+*^/*nanos*^*+*^ male PGCs rely on somatic gonadal “niche” cells for their induction.

### *klf4l*-expressing cells are present in vitellaria and are progenitors of the yolk cell lineage

Our results indicate that *klf4l* is an essential regulator of the establishment and maintenance of the planarian germ cell lineage. Is this crucial germline regulator also expressed in “somatic” organs: the vitellaria (Fig 1B)? In *S*. *mediterranea*, the vitellaria are located ventrally beneath the testes and connect to the oviducts (Fig 1A). Yolkless oocytes are fertilized in the anterior-most compartment of the oviduct (the tuba). After fertilization, zygotes are transported posteriorly through the oviducts to the genital atrium, accumulating thousands of yolk cells along the way. One or more zygotes and numerous extraembryonic yolk cells are then enclosed within egg capsules. These capsules are laid through the gonopore, and embryonic development proceeds for 2 weeks before newborn hatchlings emerge [31,55–57]. Planarian embryos rely on vitellaria-derived yolk cells for their nutritional needs and development. However, little is known about these essential reproductive structures or how yolk cells are made.

**Fig 5:**
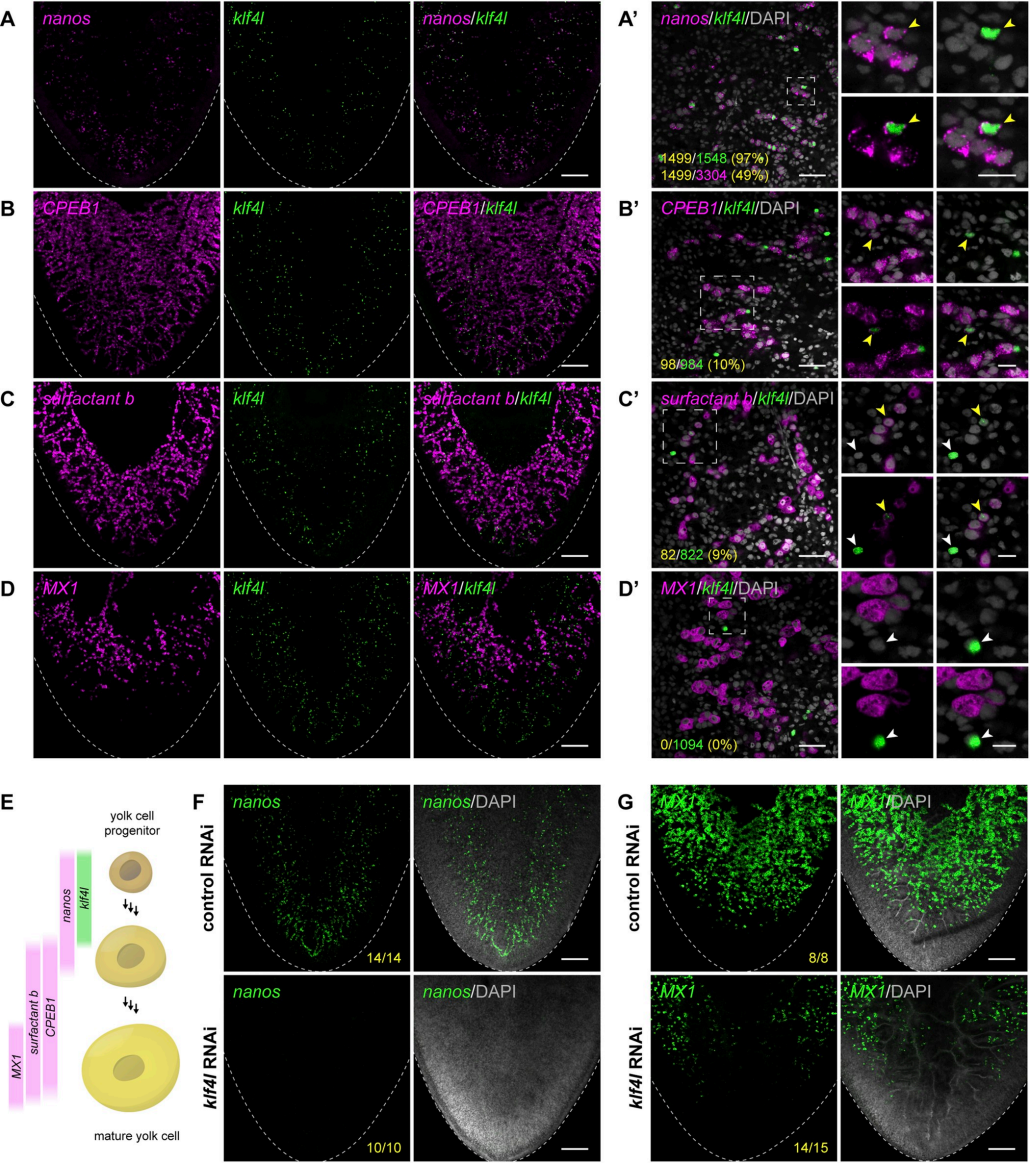
*klf4l*^*+*^ cells are present in vitellaria and are the progenitors of yolk cells. (**A–D**) Maximum intensity projections of confocal sections showing dFISH of *klf4l* (green) with *nanos* (**A**), or vitellaria markers *CPEB1* (**B**), *surfactant b* (**C**), and *MX1* (**D**) (magenta) in the ventral posterior region of sexually mature planarians. Dashed line denotes planarian boundary. (**A’–D’**) Single confocal sections of dFISH corresponding to **A–D**. (**A’**) dFISH of ventrally expressed *klf4l* (green) and *nanos* (magenta). Almost all *klf4l*^*+*^ cells coexpress *nanos* whereas *klf4l* is expressed in a subset of *nanos*^*+*^ cells. (**B’–D’**) *klf4l* is expressed in a subset of *CPEB1*^*+*^ (**B’**) and *surfactant b*^*+*^ (**C’**) yolk cells, but not in *MX1*^*+*^ yolk cells (**D’**). (**A’–D’**) Side panels are high-magnification views of outlined areas showing *klf4l* single- (white arrowheads) and double-positive cells (yellow arrowheads). Note the increase in cell size as *klf4l*^*+*^ cells differentiate into *CPEB1*^*+*^, *surfactant b*^*+*^, and ultimately *MX1*^*+*^ yolk cells. Underlying data can be found in S1 Data. (**E**) Schematic depicting genes expressed during developmental progression of yolk cell lineage. (**F, G**) Maximum intensity projections of confocal sections showing FISH of *nanos* (**F**) and *MX1* (**G**) (green) in ventral tail region of control and *klf4l* RNAi animals. Dashed line denotes planarian boundary. *N* = 3 experiments, *n* = 8 to 15 planarians. *klf4l* RNAi results in loss of *nanos*-expressing cells and a reduction of *MX1*^*+*^ yolk cells in the vitellaria. (**A’–D’, F–G**) Nuclei are counterstained with DAPI (gray). Scale bars, 200 μm (**A–D, F–G**), 50 μm for overview images, 20 μm for side panels (**A’–D’**). *klf4l*, *klf4-like*; RNAi, RNA interference.

To our surprise, ventrolateral *klf4l*-expressing cells also expressed *nanos* (97%, *n* = 1,548 *klf4l*^*+*^ cells) (Fig 5A and 5A’). Previously, *nanos* expression had only been detected in a population of eye cells and in germ cells in testes and ovaries [29,30,51]. Compared to germ cells, ventrolateral *nanos* is expressed at lower levels, but is readily detectable due to recent improvements in ISH sensitivity [58,59]. As in the gonads, ventrolateral *klf4l* expression is restricted to a subset of *nanos*^*+*^ cells (49%, *n* = 3,304 *nanos*^*+*^ cells). Are *klf4l*^*+*^/*nanos*^*+*^ cells present in the vitellaria and do they represent the progenitor of planarian yolk cells? To answer these questions and to characterize the progression of yolk cell development, we performed combinatorial dFISH analyses on mature sexual planarians to detect *klf4l* and previously reported vitellaria markers *CPEB1*, *surfactant b*, and *Monosiga brevicollis MX1 hypothetical protein* (*MX1*) [54]. We found that some *klf4l*^*+*^ cells coexpress *CPEB1* (10%, *n* = 984 *klf4l*^*+*^ cells) and *surfactant b* (9%, *n* = 822 *klf4l*^*+*^ cells), but not *MX1* (0%, *n* = 1,094 *klf4l*^*+*^ cells) (Fig 5B–5D and 5B’–5D’). These observations suggest that *klf4l* expression marks the earliest yolk cells and that *CPEB1* and *surfactant b* expression precede *MX1* expression in the yolk cell lineage. Indeed, most *CPEB1*^*+*^ cells coexpress *surfactant b* (95%, *n* = 1,752 *CPEB1*^*+*^ cells) but a much smaller fraction coexpress *MX1* (36%, *n* = 4,334 *CPEB1*^*+*^ cells) (S6A, S6B, S6A’ and S6B’ Fig). A large fraction of *surfactant b*-expressing cells are also *MX1*^*+*^ (70%, *n* = 8,057 *surfactant b*^*+*^ cells), and essentially all *MX1*^*+*^ cells coexpress *surfactant b* (99%, *n* = 5,840 *MX1*^*+*^ cells) (S6C and S6C’ Fig). The progression of yolk cell development is also marked by changes in cell morphology: *klf4l*^*+*^ cells are small and have very little cytoplasm, unlike the large, yolk-filled *surfactant b*^*+*^ and *MX1*^*+*^ cells of the vitellaria (Fig 5C’ and 5D’). Taken together, these results are consistent with a model in which *klf4l*^*+*^/*nanos*^*+*^ cells define the origin of the yolk cell lineage and that *nanos*, *CPEB1*, *surfactant b*, and *MX1* are expressed in a partially overlapping, stepwise fashion as yolk cells differentiate (Fig 5E).

To characterize the developmental origins of the vitellaria, we examined the expression patterns of *klf4l*, *nanos*, *CPEB1*, *surfactant b*, and *MX1* by dFISH at 2 earlier stages of planarian development. One-week-old hatchlings did not express any of these yolk cell markers in the presumptive vitellarial regions (*n* = 0/40 hatchlings) (S7A Fig). Later in development, 100% of 2- to 3-week-old juveniles (*n* = 26/26 planarians) possessed ventrally located *klf4l*^*+*^ cells in the region where vitellaria form (S7B Fig). Additionally, all juvenile worms expressed *nanos* (*n* = 7/7), *CPEB1* (*n* = 6/6) and *surfactant b* (*n* = 7/7) in their vitellaria, but few expressed *MX1* (*n* = 2/6) (S7B Fig). Therefore, *klf4l*^*+*^/*nanos*^*+*^ yolk cell progenitors are specified postembryonically (after hatchlings have developed into juvenile planarians), and these cells continue to differentiate into *CPEB1*^*+*^, *surfactant b*^*+*^, and ultimately *MX1*^*+*^ yolk cells as planarians mature into adults.

Is *klf4l* also required for the development of the yolk cell lineage? We induced *klf4l* RNAi beginning in hatchlings (8 to 12 feedings) and examined the effects on early and late yolk cells by FISH to detect *nanos* and *MX1*. Knockdown of *klf4l* using 3 different dsRNA constructs resulted in a dramatic reduction of all yolk cells (Figs 5F, 5G and S8), confirming the requirement for *klf4l* in the development of the yolk cell lineage and suggesting that this ventral *klf4l*^*+*^/*nanos*^*+*^ population is indeed the progenitor of yolk cells.

### Yolk cells share features with neoblasts and germ cells

**Fig 6:**
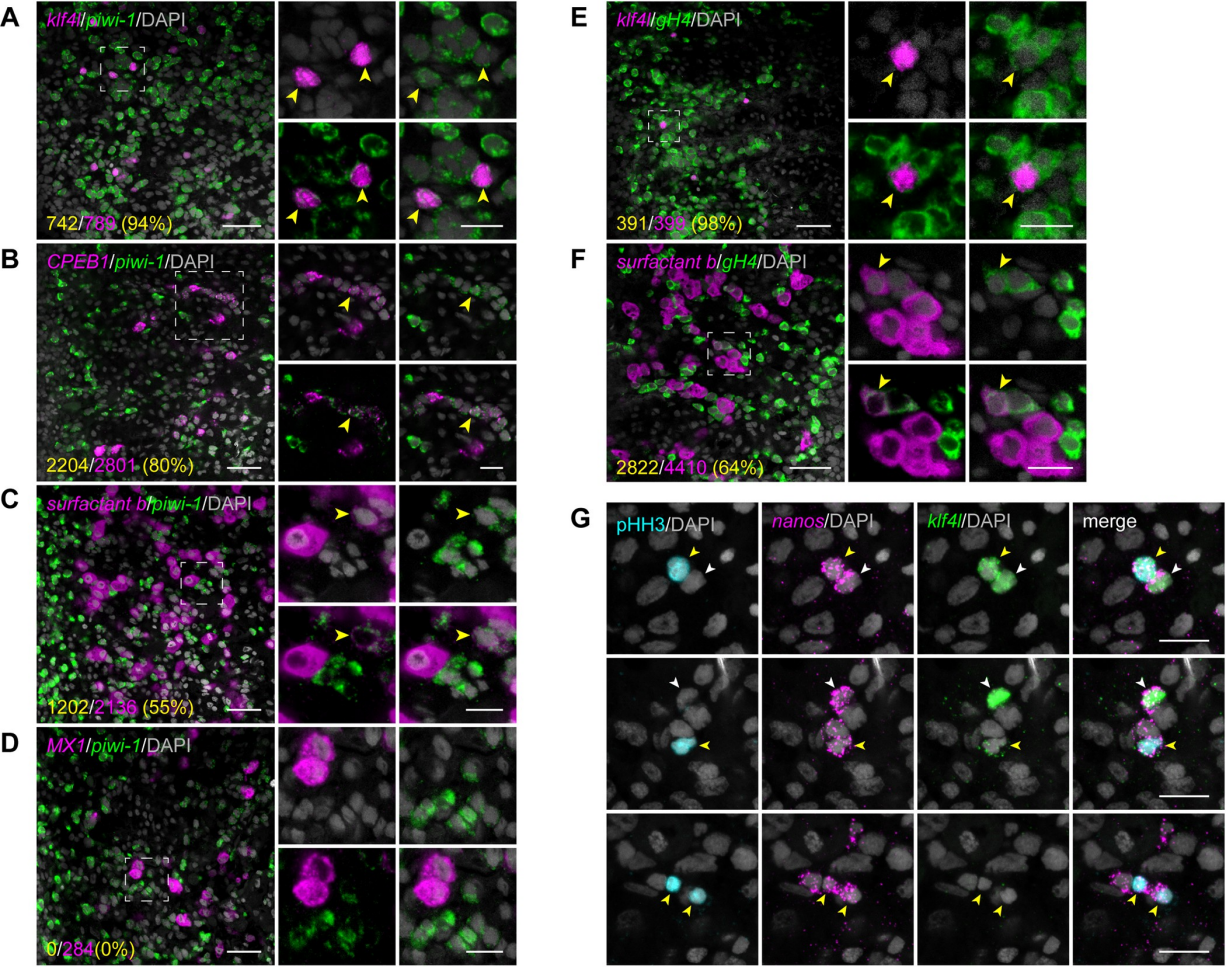
Yolk cells share features with neoblasts and germ cells. (**A–D**) Single confocal sections showing dFISH of neoblast and germ cell marker *piwi-1* (green) and *klf4l* (**A**), *CPEB1* (**B**), *surfactant b* (**C**), and *MX1* (**D**) (magenta). Side panels are high-magnification views of outlined areas showing *piwi-1* double-positive cells (yellow arrowheads). (**E, F**) Single confocal sections showing dFISH of neoblast and germ cell marker *gH4* (green) and *klf4l* (**E**) and *surfactant b* (**F**) (magenta). Side panels are high-magnification views of outlined areas showing *gH4* double-positive cells (yellow arrowheads). (**G**) Maximum intensity projections of confocal sections (5-μm thick) imaged from the ventral posterior region of sexually mature planarians showing *klf4l* (green) and *nanos* (magenta) dFISH with pHH3 (cyan) immunostaining in vitellaria. Mitotically active *klf4l*^*+*^/*nanos*^*+*^ vitellocytes with high (top panels) and low levels (middle panels) of *klf4l* expression are shown (yellow arrowheads). Nondividing *klf4l*^*+*^/*nanos*^*+*^ cells are also present (white arrowheads). *klf4l*^–^/*nanos*^*+*^ yolk cell progenitors are able to divide (bottom panels; yellow arrowheads). (**A–G**) Nuclei are counterstained with DAPI (gray). Scale bars, 50 μm for overview images, 20 μm for side panels (**A–F**), 20 μm (**G**). Underlying data can be found in S1 Data. dFISH, double FISH.

Our results suggest that *klf4l* marks the top of both germ cell and yolk cell lineages. Yolk cells are technically somatic since they do not generate gametes, yet it has long been postulated that flatworm yolk cells may share an evolutionary origin with oocytes (the female germline) [55]. One hypothesis is that yolk cells were derived from germ cells in the course of evolution and that a split/divergence between these 2 cell types may have occurred in the common ancestor of all ectolecithal flatworms [60–62]. As we found that both yolk cells and the germline share *klf4l* and *nanos* expression, we wondered whether yolk cells share other germ cell characteristics, such as expression of *piwi-1* and *germinal histone H4* (*gH4*) (S9A Fig), 2 transcripts thought to be expressed exclusively in neoblasts and germ cells [30,57,63–66]. By dFISH, we found that the vast majority of *klf4l*^*+*^ cells in the vitellaria are also *piwi-1*^*+*^ (94%, *n* = 789 *klf4l*^*+*^ cells) and *gH4*^*+*^ (98%, *n* = 399 *klf4l*^*+*^ cells) (Figs 6A, 6E, S9B, and S9C). Unlike other somatic tissues, in which *piwi-1* mRNA is degraded during differentiation [36], *piwi-1* expression perdures during yolk cell differentiation and is still detected in most *CPEB1*^*+*^ cells (80%, *n* = 2,801 *CPEB1*^*+*^ cells) and *surfactant b*^*+*^ cells (55%, *n* = 2,136 *surfactant b*^*+*^ cells), but not in *MX1*^*+*^ cells (0%, *n* = 284 *MX1*^*+*^ cells) (Fig 6B–6D). Similarly, *gH4* is coexpressed in most *surfactant b*^*+*^ cells (64%, *n* = 4,410 *surfactant b*^*+*^ cells) (Figs 6F and S9D). Thus, similar to the germ cell lineages in testes and ovaries, *piwi-1* and *gH4* expression persist in differentiating yolk cells.

In addition to the retention of germ cell features in yolk cells, these cells are mitotically active. We detected pHH3 staining in *klf4l*^*+*^/*nanos*^*+*^ as well as *klf4l*^–^/*nanos*^*+*^ yolk cells (Fig 6G). Taken together, these results show that even though yolk cells do not give rise to gametes (and are therefore not germ cells), they do exhibit several germ cell characteristics, including expression of the germline markers *nanos*, *piwi-1*, and *gH4*, and the capacity to proliferate.

### Vitellaria contain distinct cell types: A yolk cell lineage and nonyolk support cells

**Fig 7:**
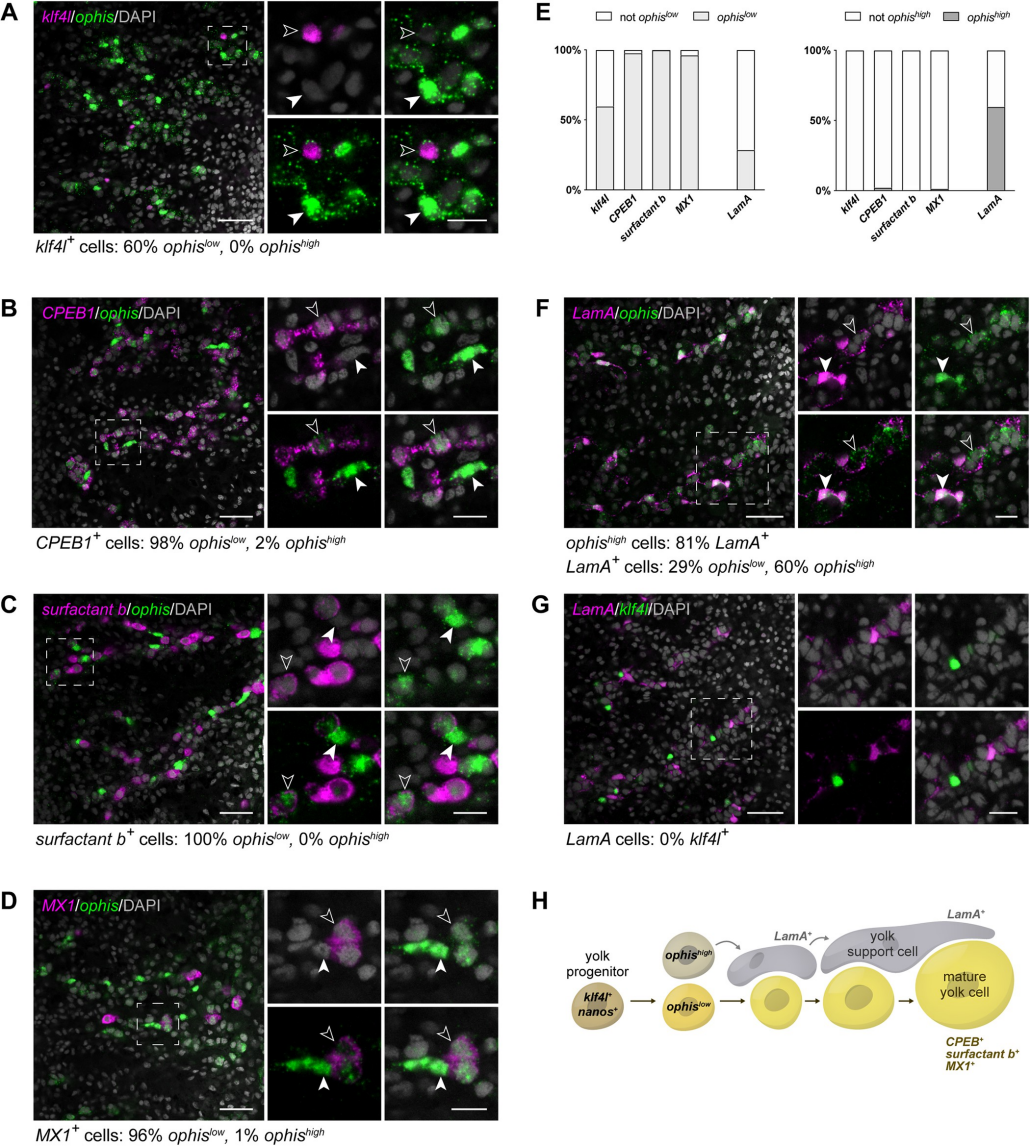
Vitellaria contain distinct cell types: Yolk cells and nonyolk support cells. (**A–D, F–G**) Single confocal sections showing dFISH. Side panels are high-magnification views of outlined areas. (**A**) dFISH of *klf4l* (magenta) and vitellaria marker *ophis* (green). *ophis*^*high*^ cells do not coexpress *klf4l* (filled white arrowhead) but *ophis*^*low*^ cells do (unfilled white arrowhead). (**B-D**) dFISH of *ophis* (green) and *CPEB1* (**B**), *surfactant b* (**C**), and *MX1* (**D**) (magenta). *ophis*^*low*^ cells (unfilled white arrowheads), but not *ophis*^*high*^ cells (filled white arrowheads), express yolk cell lineage differentiation markers. (**E**) proportion of cells in the vitellaria that coexpress low levels (left) versus high levels (right) of *ophis*. *ophis*^*low*^ cells predominantly coexpress markers of the yolk cell lineage. Conversely, most *ophis*^*high*^ cells coexpress *LamA* but do not express yolk cell markers. (**F**) dFISH of *LamA* (magenta) and *ophis* (green). *ophis*^*high*^ cells coexpress *LamA* (filled white arrowhead) whereas *ophis*^*low*^ cells do not (unfilled white arrowhead). (**G**) dFISH of *LamA* (magenta) and *klf4l* (green). *LamA* and *klf4l* are never coexpressed in the same cells. (**A–D, F–G**) Nuclei are counterstained with DAPI (gray). Scale bars, 50 μm for overview images, 20 μm for side panels. (**H**) Schematic depicting genes expressed during developmental progression of *ophis*^*low*^ yolk cells and associated *ophis*^*high*^ support cells. Underlying data can be found in S1 Data. dFISH, double FISH; *klf4l*, *klf4-like*.

Gonads are not composed solely of germ cells: They also contain somatic support cells (or niche cells) that govern germ cell behavior. Thus, we asked whether vitellaria contain nonyolk vitelline support cells and whether they could play a niche-like role in maintaining the *klf4l*^*+*^/*nanos*^*+*^ stem/progenitor population for sustaining the yolk cell lineage. Previously, expression of the orphan G-protein–coupled receptor *ophis*, a somatic gonadal cell marker, was detected in the vitellaria, but its role there was not characterized [53]. We found that in mature sexual planarians, the vitellaria are arranged in an extensively branched network containing 2 populations of *ophis*-expressing cells: *ophis*^*high*^ cells, which express *ophis* predominantly in the nucleus, and *ophis*^*low*^ cells with weak signal throughout the cell (Fig 7A). *ophis*^*+*^ cells are interspersed throughout the vitellaria, similar to *klf4l*^*+*^ cells (S10A–S10D Fig). *klf4l*^*+*^ cells are tightly juxtaposed with *ophis*^*high*^ cells; however, they never coexpress high levels of *ophis* (0% *klf4l*^*+*^ cells are *ophis*^*high*^, *n* = 368 *klf4l*^*+*^ cells) (Fig 7A and 7E). On the other hand, a large fraction of *klf4l*^*+*^ cells are *ophis*^*low*^ (60% *klf4l*^*+*^ cells are *ophis*^*low*^, *n* = 368 *klf4l*^*+*^ cells). These results led us to hypothesize that *ophis*^*low*^ versus *ophis*^*high*^ cells represent 2 distinct classes of cells in the vitellaria: *ophis*^*low*^ cells constitute the yolk cell lineage proper and *ophis*^*high*^ cells are support cells that closely associate with the yolk cells and comprise the remaining structure of the vitellaria.

If the *ophis*^*low*^ population represents the yolk cell lineage of which *klf4l*^*+*^ cells are the precursors, then we would expect *klf4l*^–^/*ophis*^*low*^ cells to express markers of progressive yolk cell differentiation. Consistent with this idea, almost all *CPEB1*^*+*^, *surfactant b*^*+*^, and *MX1*^*+*^ cells coexpress low levels of nuclear and cytoplasmic *ophis* mRNA (98%, *n* = 2,914 *CPEB1*^*+*^ cells; 100% *n* = 1,760 *surfactant b*^*+*^ cells; 96%, *n* = 256 *MX1*^*+*^ cells) (Fig 7B–7E). On the other hand, high levels of nuclear *ophis* were rare in *CPEB1*^*+*^, *surfactant b*^*+*^, and *MX1*^*+*^ cells (2%, *n* = 2,914 *CPEB1*^*+*^ cells; 0%, *n* = 1,760 *surfactant b*^*+*^ cells; 1%, *n* = 256 *MX1*^*+*^ cells). These results indicate that *ophis*^*low*^ expression emerges in a subset of *klf4l*^*+*^ yolk cell progenitors and subsequently persists as these cells differentiate (Fig 7H), whereas *ophis*^*high*^ expression defines a distinct cell type in the vitellaria.

In agreement with the model that *ophis*^*high*^ cells constitute a separate cell lineage, the majority of these cells do not express yolk cell markers (0%, *n* = 784 *ophis*^*high*^ cells are *klf4l*^*+*^; 12%, *n* = 519 *ophis*^*high*^ cells are *CPEB1*^*+*^; 0%, *n* = 721 *ophis*^*high*^ cells are *surfactant b*^*+*^; 1%, *n* = 521 *ophis*^*high*^ cells are *MX1*^*+*^). Instead, most *ophis*^*high*^ cells express *Laminin A* (*LamA*) (81%, *n* = 440 *ophis*^*high*^ cells are *LamA*^*+*^) (Fig 7F), a gene expressed in the vitellaria (S10E and S10F Fig) as well as in somatic gonadal cells in the testes and ovaries (S10G Fig). This result corroborates the finding that *ophis*^*high*^ expression marks support cells within the vitellaria. Notably, *klf4l* and *LamA* are never coexpressed within the vitellaria (0%, *n* = 540 *klf4l*^*+*^ cells, 0%, *n* = 867 *LamA*^*+*^ cells) (Fig 7G). Taken together, our data suggest that 2 cell lineages exist in the vitellaria: the yolk cell lineage (*ophis*^*low*^), which includes *klf4l*^*+*^ cells, and a second population made up of *ophis*^*high*^/*LamA*^*+*^ cells. It was previously reported that *ophis* transcript was expressed in the somatic gonadal cells of the ovary [53]. In addition to this expression pattern, we detect low levels of *ophis* expression in the oogonial lineage, similar to yolk cells (S10H Fig). The dichotomy between *ophis*^*low*^ versus *ophis*^*high*^ expression in the germline and somatic lineages of the ovary is reminiscent of what we observed in the 2 vitellarial lineages.

### Gonadal niche factor *ophis* is required to maintain the yolk cell lineage

**Fig 8:**
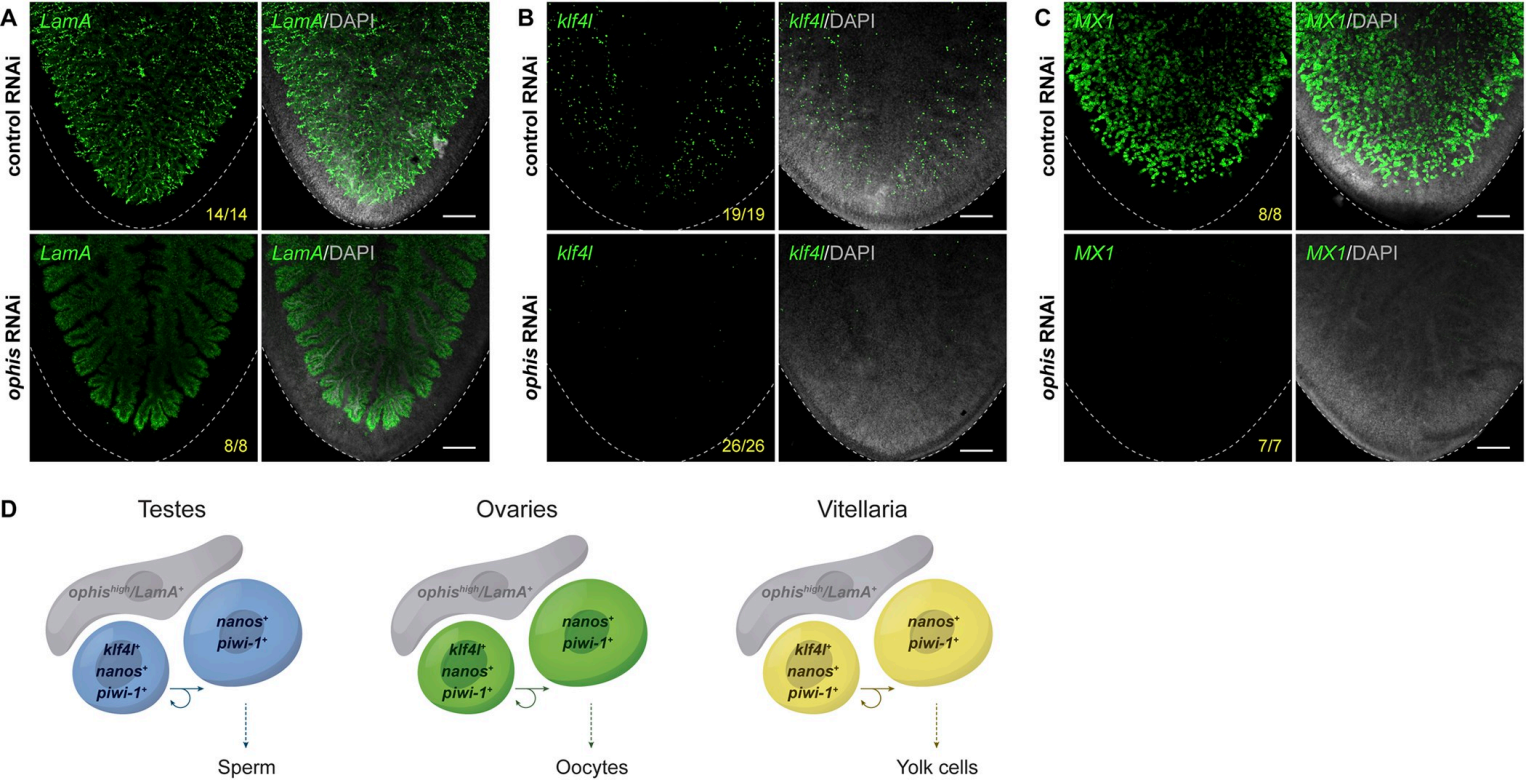
Germ cell niche factor *ophis* is required to sustain yolk cell production/vitellogenesis. (**A–C**) Maximum intensity projections of confocal sections showing FISH of *LamA* (**A**), *klf4l* (**B**), and *MX1* (**C**) (green) in the ventral posterior region of sexually mature control versus *ophis* RNAi animals. Dashed line denotes planarian boundary. *N* = 3 to 5 experiments, *n* = 7 to 26 planarians. (**A**) *ophis* RNAi results in a dramatic loss of the *LamA*^*+*^ cells throughout the vitellaria. Note that *LamA* expression is only visible in the branched gut in *ophis* RNAi planarians. (**B**, **C**) *ophis* RNAi results in a reduction of *klf4l*^*+*^ yolk cell progenitors and *MX1*^*+*^ differentiated yolk cells. (**A–C**) Nuclei are counterstained with DAPI (gray). Scale bars, 200 μm. (**D**) Model depicting similarities shared between gonads (where gametogenesis occurs) and vitellaria (where yolk cell production occurs). *klf4l*^*+*^/*nanos*^*+*^/*piwi-1*^*+*^ presumptive GSCs in testes and ovaries divide and give rise to *klf4l*^–^/*nanos*^*+*^/*piwi-1*^*+*^ progeny. These germ cells are supported by *ophis*^*+*^ somatic gonadal niche cells. Vitellaria are comprised of *klf4l*^*+*^/*nanos*^*+*^/*piwi-1*^*+*^ “germ cell–like” yolk progenitors that are mitotically competent, sustain yolk cell production, and are supported by *ophis*^*high*^ support cells. FISH, fluorescent RNA in situ hybridization; *klf4l*, *klf4-like*; RNAi, RNA interference.

Previous work has shown that *ophis* is required for proper development of both male and female gonads in planarians [53]. To address whether *ophis* is a shared molecular regulator of gonads and vitellaria, we performed RNAi knockdown of *ophis* in hatchlings until they reached sexual maturity and analyzed the effects on the vitellaria by FISH (Fig 8A–8C). *ophis* knockdown resulted in a dramatic loss of the *LamA*^*+*^ cells throughout the vitellaria, but did not affect *LamA* expression within the gut (Fig 8A). We also observed a significant reduction of *klf4l*^*+*^ cells and complete loss of mature *MX1*^*+*^ yolk cells in *ophis* RNAi animals (Fig 8B and 8C). Although we cannot distinguish the functions of *ophis*^*high*^ from *ophis*^*low*^ cells in the vitellaria by available techniques in planarians, it is clear from our data that *ophis* is essential for the maintenance of support cells (*ophis*^*high*^/*LamA*^*+*^) in the vitellaria and is required (perhaps through the action of support cells) for the maintenance and differentiation of *klf4l*^*+*^ yolk cell progenitors.

## Discussion

Most animals specify PGCs and segregate them from somatic tissues only once, early in development. Within developed gonads, germ cells are generated from GSCs for the reproductive life of the organism. Planarians also specify PGCs in development but are able to continuously regenerate new germ cells from pluripotent stem cells throughout their lifetime. Whether or not planarians also maintain GSCs is less clear, especially since theoretically they could reseed gonads with new germ cells from their somatic stem cells (neoblasts) throughout adulthood. Characterizing the regulators that define planarian germ cells and function in their specification and maintenance will reveal important clues for understanding the remarkable ability of planarians to faithfully regenerate germ cells.

### Elucidating early stages of the germ cell lineage

We found that *klf4l* expression marks the earliest/least differentiated germ cell state in planarians. Early hatchlings specify PGCs that coexpress *klf4l* and *nanos* dorsolaterally, where adult testes will ultimately reside. Thus, male PGCs likely do not undergo extensive migration to the somatic gonad. Instead, they are likely to be specified along with *dmd1*-expressing somatic gonadal niche cells and then differentiate in situ as the testis grows/elaborates during reproductive maturation. As hatchlings develop into juveniles, testis primordia grow in size and 2 successive populations of *klf4l*^–^ germ cells arise (*nanos*^*+*^ and *nanos*^–^). These populations emerge as testes develop, strongly suggesting that they represent the first steps in germ cell differentiation and that cessation of *klf4l* expression may be required for germ cell differentiation to proceed.

We observed that *klf4l* is expressed in virtually all *nanos*^*+*^ cells in early hatchlings and then becomes increasingly restricted to a smaller subset of *nanos*^*+*^ cells as planarians sexually mature. The restriction of *klf4l* expression to a subset of *nanos*^*+*^ germ cells holds true in asexual planarians as well, where the number of germ cells in both gonadal primordia increases as animals grow [29]. Small asexual planarians express *klf4l* in almost all *nanos*^*+*^ germ cells, whereas larger asexuals have proportionally fewer double-positive cells in their testis primordia. Our results refine the stage at which development arrests in asexuals: in growing asexuals, *klf4l*^*+*^/*nanos*^*+*^ cells can only carry out the first step of development (into *klf4l*^–^/*nanos*^*+*^ cells), further reinforcing the idea that the germ cell lineage progresses in this direction.

Additionally, we have shown that *klf4l* is required for the specification of germ cells. RNAi knockdown of *klf4l* in soma-only head fragments results in regenerated animals that do not respecify *nanos*^*+*^ germ cells, even though new testis somatic gonadal support cells (*dmd1*^*+*^) that form the niche are made. Our data suggest that *klf4l* is required cell autonomously for the de novo specification of germ cells. Taken together, these observations support a model in which *klf4l* expression marks the top of the germ cell hierarchy and that expression of *klf4l* is required for the acquisition of germ cell fate.

Although animals specify their germline in different ways (preformation versus induction), a conserved feature of newly specified PGCs is the repression of somatic differentiation transcriptional programs. Posttranscriptional regulation through the action of conserved germline-specific RNA regulators such as *vasa*, *pumilio*, *nanos*, and *piwi* plays an outsized role in controlling germ cell fate, survival, proliferation, and differentiation. Germ cell fate specification at the transcriptional level is less well understood. During mouse embryogenesis, PGCs are specified from pluripotent epiblast cells by BMP signals from the extraembryonic ectoderm and the visceral endoderm through the action of Smads [67–70]. Critical regulators of PGC specification have been described, including transcription factor genes *Prdm1* (which encodes BLIMP1) and *Prdm14* [71–78]. A key role of BLIMP1 is to induce expression of *Tcfap2c* (which encodes the transcription factor AP2γ) [75,79,80], and together, BLIMP1, PRDM14, and AP2γ are important for initiating PGC specification, repressing expression of somatic genes, activating expression of PGC-specific genes, and driving epigenetic reprograming [78–82].

Recent studies on emerging models have shown that some of the molecular mechanisms regulating PGC specification may be conserved. In the cricket *Gryllus bimaculatus*, PGCs are specified in response to BMP signaling via the action of Blimp-1 [83,84]. Additionally, in the cnidarian *Hydractinia symbiolongicarpus*, a homolog of AP2 is an inducer of germ cell fate [24]. Although the inductive cues that control germ cell fate in *S*. *mediterranea* remain to be identified, here, we identify a transcription factor, Klf4l, required for germ cell specification. It is worth noting that Klf4 is a crucial pluripotency factor in mammals. Furthermore, pluripotency genes *Oct4*, *Sox2*, and *Nanog* are expressed in mouse PGCs [85], reflecting the importance of maintaining pluripotency in germ cells. Therefore, future identification of Klf4l targets in *S*. *mediterranea* will not only elucidate the transcriptional program required for promoting germ cell fate from pluripotent neoblasts but may also provide important clues into how germline pluripotency is maintained.

### Are *klf4l*-expressing cells true stem cells?

GSCs are characterized by the ability to undergo self-renewing divisions in which one daughter remains a stem cell and the other differentiates. Consistent with the hypothesis that *klf4l*^*+*^ cells are GSCs, *klf4l*-expressing cells in both testes and ovaries are mitotically active throughout postembryonic development. However, technical limitations precluded us from testing whether mitotic *klf4l*^*+*^ cells undergo self-renewing divisions. Alternatively, it is possible that no resident GSC population exists within the gonads themselves; instead, neoblasts residing in the gonad-adjacent parenchyma may be continually specified as new germ cells that then differentiate directly without self-renewing. Either way, dFISH experiments with *klf4l* and *nanos* have uncovered heterogeneity within the early male and female germ cell compartments. Furthermore, developmental timeline experiments have allowed us to define the early germ cell lineage with *klf4l*^*+*^/*nanos*^*+*^ germ cells at the top of the hierarchy. Prolonged inhibition of *klf4l* via RNAi during postembryonic development and sexual maturation led to a dramatic loss of early germ cells in both testes and ovaries, resulting in agametic gonads in some animals. This result suggests that *klf4l* is required for the maintenance of GSCs (or germ cell lineal progenitors) to sustain gametogenesis. Future experiments will explore whether *klf4l*^*+*^/*nanos*^*+*^ cells represent true GSCs and whether this newly defined lineage progresses in a unidirectional manner, or if all *nanos*-expressing cells retain GSC-like potential.

### Bidirectional soma-germ cell communication in the ovary

Intriguingly, loss of germ cells in the ovary led to a corresponding increase in ovarian somatic gonadal cells (*ophis*^*+*^ and *LamA*^*+*^). This result reveals that soma-germline communication in the planarian ovary is bidirectional. The importance of somatic support cells for germ cell development is undisputed. However, far less is known about how germ cells signal back to their somatic microenvironment [86–88]. In planarians, somatic cell expansion in the ovary in response to germ cell loss suggests that somatic and germ cell numbers are coordinated via a feedback mechanism. What signals regulate this feedback loop? How does the planarian ovary balance somatic and germ cell numbers to achieve an equilibrium between these 2 cell types? The planarian ovary presents a unique opportunity to investigate the mechanisms involved in soma-germline coordination during development, homeostasis, and regeneration.

While both gonads contain *klf4l*^*+*^/*nanos*^*+*^ putative GSCs, there is also a population of these cells anterior to each ovary. They may be germ cell progenitors that migrate posteriorly and enter the ovary, where they then give rise to *nanos*^*−*^ oogonia/oocytes. Alternatively, *klf4l*^*+*^/*nanos*^*+*^ cells may be specified in a permissive zone along the medial posterior regions of the cephalic ganglia, but only the posterior-most germ cells located at the base of the brain (where the somatic gonad is located) are then able to associate with somatic gonadal cells and consequently instructed to differentiate. Until we are able to specifically ablate this population, its contribution to the ovary (or lack thereof) will remain mysterious.

### A shared evolutionary origin of germ cells and yolk cells?

A unique reproductive feature of flatworms is ectolecithality: a developmental novelty in which oocytes develop with little/no yolk while specialized yolk cells are produced ectopically. For embryogenesis to occur, the fertilized oocyte and numerous yolk cells must be deposited together in egg capsules. As yolk cells are the sole source of embryonic nutrients, ectolecithality has led to marked evolutionary and functional consequences on embryonic development. For example, yolkless embryos develop temporary organs (e.g., embryonic pharynx and primitive gut) that facilitate uptake of maternally provided yolk/nutrients early in embryogenesis [89].

Recent phylogenetic analyses have shed light on the origin of ectolecithality in flatworms. One group of flatworms produces oocytes and yolk cells within a single organ (the germovitellarium); another group partitions egg- and yolk cell-production into 2 distinct organs (the germarium/ovary and vitellaria). This latter group is known as Euneoophora and includes planarians and parasitic flatworms. Although traditional phylogenies grouped both types of ectolecithal worms together, recent phylogenies suggest that they evolved independently [60–62]. Thus, the ectolecithal common ancestor of all euneoophorans likely evolved from more primitive endolecithal (“yolky egg”-producing) flatworms [61,62], consistent with a model in which yolk cells in ectolecithal flatworms evolved from ancestral “yolky” germ cells. These phylogenetic studies recognized that molecular similarities between germ cell and yolk cell precursors would lend further support to the shared evolutionary origin hypothesis [60–62]. Here, we provide molecular and developmental evidence suggesting that yolk cells and germ cells are homologous. Even though yolk cells do not produce gametes and, therefore, are not de facto germ cells, they share several molecular and cellular characteristics in common with germ cells (Fig 8D). Yolk cells express both *klf4l* and *nanos*: 2 markers that define male and female germ cell lineages. Similarly to testes and ovaries, *klf4l* expression in vitellaria is restricted to a subset of *nanos*^*+*^ yolk cells, suggesting that *klf4l*^*+*^/*nanos*^*+*^ cells define the lineal progenitors of yolk cells. We also find that yolk cells express *piwi-1* and *gH4*, which until this work, were reported to be expressed exclusively in neoblasts and germ cells. *piwi-1* and *gH4* are highly expressed in neoblasts but downregulated in their immediate somatic progeny. In contrast to the soma, but similar to *piwi-1* and *gH4* expression in male and female germ cell lineages, expression of these genes is sustained in differentiating (*klf4l*^–^/*nanos*^–^) yolk cells. This sustained expression of neoblast/germ cell markers provides another molecular similarity between germ cells and yolk cells.

Surprisingly, we observed mitosis in yolk cells. Previously, the only planarian somatic cells thought to have mitotic activity were neoblasts. Although yolk cells are technically somatic, our results clearly indicate that like germ cells, a subset of yolk cells is mitotically competent. The observation that both *klf4l*^*+*^/*nanos*^*+*^ and *klf4l*^–^/*nanos*^*+*^ yolk cells divide indicates that mitotic ability is not limited to the earliest progenitor in the yolk cell lineage. Are pHH3^+^/*klf4l*^*+*^/*nanos*^*+*^ cells undergoing self-renewing divisions? Do *piwi-1*^*+*^/*klf4l*^*+*^/*nanos*^*+*^ cells represent a new stem cell population in planarians? With the sole exception of planarian gonads, no other planarian organ contains a resident stem cell (or dividing cell) population. Instead, dividing neoblasts in the parenchyma are the only source of new differentiated somatic cells, which then integrate into existing tissues. The planarian vitellarium provides an intriguing case study to understand the regulation of stem cell populations in planarians.

These similarities between the female germ cell and yolk cell lineages prompted us to ask whether ovaries and vitellaria also share structural features. For example, is there a distinct lineage of somatic support cells that acts as a niche? Gonads are typified by the presence of somatic support cells that associate intimately with germ cells and play crucial roles in their development. We discovered that in addition to the yolk cells, vitellaria contain a second population of cells (*ophis*^*high*^/*LamA*^*+*^) with long fingerlike projections that contact all stages of the yolk cell lineage. Both *ophis* and *LamA* are also expressed in the somatic gonadal cells of the ovary. *ophis* RNAi leads to loss of *LamA*^*+*^ vitellaria cells, a dramatic decrease in *klf4l*^*+*^ yolk cell progenitors, and a complete failure of vitellogenesis, suggesting that the *ophis*^*high*^/*LamA*^*+*^ cells could function as a niche required to maintain the yolk cell stem/progenitor population. Because a significant number of *klf4l*^*+*^ yolk cell progenitors coexpress low levels of *ophis*, we cannot yet distinguish definitively between a cell-autonomous versus nonautonomous role for *ophis* in yolk cell development. However, since *ophis* RNAi results in a dramatic loss of *klf4l*^*+*^ cells that far outnumbers the fraction of *klf4l*^*+*^ cells that coexpress *ophis* (60%), we favor the model that *ophis* acts nonautonomously in the maintenance of *klf4l*^*+*^/*ophis*^*−*^ yolk cells.

Comparative analyses of gametogenesis and vitellogenesis in *S*. *mediterranea* have allowed us to investigate the biological phenomenon of ectolecithality and to better understand its origin in Platyhelminthes. Interestingly, *nanos* expression has been detected in early yolk cells of the parasitic flatworm *Schistosoma mansoni* [47]. Since *all* parasitic flatworms (trematodes, cestodes, and monogeneans) are characterized by the presence of ectolecithality, and depend on sexual reproduction to successfully propagate, the vitellaria may provide an effective anti-helminthic target. Thus, the experimental accessibility of planarians provides an opportunity to dissect the mechanisms regulating vitellaria development, with the potential to help in the fight against their parasitic cousins.

## Conclusions

This study demonstrates the functional requirement for *klf4l* in germ cell specification and maintenance in planarians and provides evidence that *klf4l* expression marks the top of the germ cell lineage. Additionally, our results suggest that *klf4l* is a pivotal intrinsic regulator not only of germ cells, but also of yolk cells in a somatic reproductive structure, the vitellaria. Furthermore, we identify a new population of mitotically competent yolk cell progenitors and characterize their niche. Together, these results show that planarian germ cells and somatic yolk cells exhibit a remarkable degree of similarity, supporting the hypothesis that these 2 lineages share an evolutionary origin.

## Materials and methods

### Planarian culture

Sexual *S*. *mediterranea* [63] were maintained in 0.75X Montjuïc salts [90] at 16 to 18°C. Asexual *S*. *mediterranea* (clonal strain CIW4) [91] were maintained in 1X Montjuïc salts at 20 to 22°C. Planarians were starved for 1 week before experimentation.

### Cloning

Target genes were cloned by PCR amplification of cDNA generated from RNA extracted from adult sexual *S*. *mediterranea*. Gene-specific PCR amplicons were ligated into the pJC53.2 vector via TA-cloning as previously described [65]. Anti-sense riboprobes were generated by in vitro transcription reactions with T3 or SP6 RNA polymerases [59]. dsRNA was generated using T7 RNA polymerase [92]. Sequences used for probes and dsRNA are found in S1 Table.

### In situ hybridization

FISH protocols were performed as previously described [58,59] with the following modifications. Asexual and sexual hatchling/sexual adult planarians were killed in 7.5% N-acetyl-L-cysteine in 1X PBS for 5/10 minutes; fixed in 4% formaldehyde in PBSTx (1X PBS + 0.1% Triton X-100) for 15/30 minutes; bleached in Bleaching Solution (1X SSC solution containing 5% deionized formamide and 1.2% hydrogen peroxide) for 2/4 hours; incubated in PBSTx containing 10 μg/ml Proteinase K and 0.1% SDS for 10/20 minutes; and refixed in 4% formaldehyde in PBSTx for 10/15 minutes. Planarians were blocked in Blocking Solution (5% heat inactivated horse serum, 5% Roche Western Blocking Buffer in TNTx [0.1 M Tris pH 7.5, 0.15 M NaCl, 0.3% Triton X-100]) for 2 hours at room temperature, and incubated in Blocking Solution containing anti-Digoxigenin-POD (1:2,000), anti-Fluorescein-POD (1:2,000), or anti- Dinitrophenyl-HRP (1:2,000) for 8 hours at 12°C. For fluorescent development of riboprobes, TSA reactions were performed for 30 minutes.

### pHH3 immunofluorescence

Immunostaining was performed after FISH development by reblocking planarians in Blocking Solution (5% heat inactivated horse serum, 5% Roche Western Blocking Buffer in TNTx) for 2 hours at room temperature, labeling mitotic cells with anti-phospho-Histone H3 (Ser10) (1:2,000) in Blocking Solution overnight at 12°C, washing 6X in PBSTx (30 minutes each), reblocking for 2 hours at room temperature, and incubating with HRP-conjugated goat anti-mouse (1:500) in blocking solution overnight at 12°C. Planarians were washed 6X in PBSTx (30 minutes each) and TSA was performed for 30 minutes.

### Imaging

Confocal imaging was performed using a ZEISS LSM 880 with the following objectives: EC Plan-Neofluar 10x/0.3 M27, Plan-Apochromat 20x/0.8 M27, Plan-Apochromat 40x/1.3 Oil DIC M27. Image processing was performed using ZEISS ZEN 3.1 (blue edition) for linear adjustments and maximum intensity projections.

### RNA interference

In vitro dsRNA synthesis was performed as previously described [92] by in vitro transcription from PCR amplicons flanked by T7 promoters. In vitro transcription reactions were carried out overnight at 31°C, DNase-treated, brought up to 80 to 100 μl final volume with water, and annealed. dsRNA was added to liver (1:2–1:5) and fed to animals. dsRNA generated from the *CamR* and *ccdB*-containing insert of the pJC53.2 vector was used for all control RNAi feedings [65].

### Quantification and statistical analysis

Z-stack images through ovaries, testes, and vitellaria were visualized on ZEISS ZEN 3.1 (blue edition) or Imaris (Oxford Instruments, UK, Bitplane AG, Switzerland) software and cell counting was performed manually. Binary decisions (positive/negative or high/low) were made for cells using single color channels at a time. Counts for all experiments are detailed in S1 Data. Statistical analyses were performed using GraphPad Prism software. Statistical tests, significance levels, number of data points, cell or planarian numbers (*n*), and experimental replicates (*N*) are provided in the text and/or figure legends.

### Quantitative PCR

Total RNA was extracted using TRIzol Reagent (Invitrogen, Thermo Fisher Scientific, USA) from whole sexual animals 1 week after the final dsRNA feeding. Total RNA was DNase-treated and purified, followed by cDNA synthesis using the iScript cDNA Synthesis Kit (Bio-Rad Laboratories, Inc., USA). *klf4l* primers were designed to sequences outside of the sequence targeted by the dsRNA in order to avoid spurious amplification of dsRNA along with cDNA. For example, primers to the amino terminus of *klf4l* were used to quantify *klf4l* knockdown levels in *klf4l* RNAi (carboxyl terminus) animals in which dsRNA targeting the carboxyl terminus was used, and vice versa. All quantitative PCR (qPCR) analyses were normalized to endogenous control gene *β-tubulin*. qPCR primers are listed in S2 Table. qPCR was performed using the GoTaq qPCR Master Mix reagent system (Promega, USA) on a StepOnePlus Real-Time PCR System (Applied Biosystems, Thermo Fisher Scientific, USA). Three technical replicates were run for each of 3 biological replicates. ΔΔCt calculations were performed in Microsoft Excel and 2^−ΔΔCt^ values (normalized to control RNAi) with 95% confidence intervals were plotted using GraphPad Prism software.

## Supporting information

S1 Fig The Klf gene family in *S*. *mediterranea*. (**A**) The *S*. *mediterranea* genome contains 5 genes that encode Klf proteins [93]. Alignment of *S*. *mediterranea* Klf DBDs. The height of the red bars reflects percent conservation of the amino acid in the alignment. Sequence logo at bottom of the alignment depicts the consensus sequence. All Klfs are characterized by exactly 3 highly conserved C2H2 ZnF domains (each one outlined by a box) separated by intervening linker sequences and located at the carboxyl terminus. The black arrow points to a canonical aspartic acid residue (D) in ZnF2, which is important for Klf-DNA binding and is conserved in all filozoa (animals and their nearest unicellular relatives) [94]. Note the additional 5 amino acids in ZnF2 in *S*. *mediterranea* Klf4l (SMEST031008001.1.protein). These additional 5 residues in ZnF2 are a triclad innovation and are present in Klf4l homologs in the Platyhelminthes *S*. *mediterranea* (Smed), *Schmidtea polychroa* (Spol), *Dugesia japonica* (Djap), *Dendrocoelum lacteum* (Dlac), *Planaria torva* (Ptor), *Polycelis nigra* (Pnig), and *Polycelis tenuis* (Pten) (all from the Tricladida order), but not in *Prostheceraeus vittatus* (Pvit) and *Stylochus ellipticus* (Sell) (Polycladida). (**B**) The most significant human and mouse BLASTP hits for *S*. *mediterranea* Klf4l are Klf4 proteins. Alignment of *Homo sapiens* (NP_004226.3), *Mus musculus* (NP_034767.2), *Xenopus tropicalis* (NP_001017280.1), and *Danio rerio* (NP_571798.1) Klf4 DBDs with *S*. *mediterranea* (SMEST031008001.1) Klf4l DBD. DBD, DNA-binding domain; *klf4l*, *klf4-like*; ZnF, zinc finger.
(PDF)

S2 Fig *klf4l* is expressed in a subset of *nanos*^*+*^ female germ cells in sexual and asexual planarians. (**A**, **B**) Confocal section showing triple FISH of *piwi-1* (cyan), *klf4l* (green), and *nanos* (magenta) in female germ cells in hatchlings and sexually mature ovary. *klf4l* is expressed in a subset of *nanos*^*+*^/*piwi-1*^*+*^ female germ cells (compare filled (*klf4l*^*+*^) to unfilled (*klf4l*^–^) yellow arrowhead). All *klf4l*^*+*^/*nanos*^*+*^ germ cells are *piwi-1*^*+*^. A small fraction of *klf4l*^–^*/nanos*^*+*^ cells do not express *piwi-1* and are not germ cells (white arrowhead). (**C**, **D**) Confocal sections showing dFISH of *klf4l* (green) and *nanos* (magenta) in female germ cells (located mediolaterally along the planarian brain) in small (**C**) and large (**D**) asexual planarians. *klf4l* is expressed in a subset of *nanos*^*+*^ female germ cells. Insets show high-magnification views of heterogeneity of *klf4l* expression in *nanos*^*+*^ cells. (**A–D**) Percentages reflect *nanos*^*+*^ germ cells that are also *klf4l*^*+*^. Nuclei are counterstained with DAPI (gray). Scale bars, 100 μm (**A**, **B**), 50 μm for whole-brain images, 10 μm for insets (**C, D)**. Underlying data can be found in S1 Data. FISH, fluorescent RNA in situ hybridization; *klf4l*, *klf4-like*.
(PDF)

S3 Fig *klf4l* is required for oogenesis and restricts expansion of somatic gonadal cells in adult ovaries. (**A**) Single confocal section of an ovary located at the posterior of the brain (br) and anterior to the tuba/oviduct (tu/od) showing dFISH of *LamA* (magenta; somatic gonadal cells) and *CPEB1* (green; oocytes) in control and *klf4l* RNAi planarians. *klf4l* RNAi leads to oocyte loss and a nonautonomous increase in somatic support cells. Nuclei are counterstained with DAPI (gray). Scale bars, 100 μm. (**B**) Quantification of newly specified PGCs in head regenerates. Data are presented as mean ± SD. *N* = 3 to 5 experiments, *n* = 14 to 35 planarians. *p* < 0.0001, Welch ANOVA test. Underlying data can be found in S1 Data. dFISH, double FISH; *klf4l*, *klf4-like*; RNAi, RNA interference.
(PDF)

S4 Fig Quantification of *klf4l* expression in knockdown animals. qPCR analysis of *klf4l* mRNA expression (normalized to *β-tubulin*) in control and *klf4l* RNAi animals depicting efficient knockdown of *klf4l* after RNAi treatment. Top: dsRNA targeting the amino terminus of *klf4l* was used for RNAi-mediated knockdown of *klf4l*, and qPCR primers targeting the carboxyl terminus were used to quantify *klf4l* expression levels. Bottom: dsRNA targeting the carboxyl terminus of *klf4l* was used for RNAi and qPCR primers targeting the amino terminus were used to quantify *klf4l* expression levels. *N* = 3 biological replicates (3 technical replicates each). Bar graphs depict 2^−ΔΔCt^ values (normalized to control RNAi) with 95% confidence intervals. Underlying data can be found in S1 Data. *klf4l*, *klf4-like*; qPCR, quantitative PCR; RNAi, RNA interference.
(PDF)

S5 Fig Independent RNAi triggers targeting nonoverlapping *klf4l* sequences result in similar phenotypes in the gonads. (**A**) Single confocal section of an ovary located posterior to the brain (br) and anterior to the tuba/oviduct (tu/od) showing dFISH of *LamA* (magenta; somatic gonadal cells) and *CPEB1* (green; oocytes) in control and *klf4l* RNAi planarians. RNAi resulting from dsRNA targeting near-full-length *klf4l*, an amino-terminal portion of *klf4l*, or a carboxyl-terminal portion of *klf4l* all lead to similar defects in oogenesis. *N* = 2 experiments, *n* = 11–16 planarians. (**B**) Maximum intensity projections of confocal sections showing dFISH of *dmd1* (magenta; somatic gonadal cells) and *nanos* (green) in a dorsal tail region where testes reside. All *klf4l* knockdowns lead to similar defects in spermatogenesis and “collapsed” testes due to male germ cell loss. Dashed line denotes planarian boundary. *N* = 2 experiments, *n* = 10 to 14 planarians. (**A**, **B**) Nuclei are counterstained with DAPI (gray). Scale bars, 50 μm (**A**), 200 μm (**B**). dFISH, double FISH; *klf4l*, *klf4-like*; RNAi, RNA interference.
(PDF)

S6 Fig Defining the stages of yolk cell development. (**A–C**) Maximum intensity projections of confocal sections showing dFISH of vitellaria markers *CPEB1* (**A-B**), *surfactant b* (**A, C**), and *MX1* (**B, C**) in the ventral posterior region of sexually mature planarians. Dashed line denotes planarian boundary. (**A’–C’**) Single confocal sections of dFISH corresponding to **A-C**. (**A’**) dFISH of ventrally expressed *CPEB1* (magenta) and *surfactant b* (green). Almost all *CPEB1*^*+*^ cells coexpress *surfactant b* and all *surfactant b*^*+*^ cells are *CPEB1*^*+*^. (**B’**) dFISH of *CPEB1* (magenta) and *MX1* (green). A subset of *CPEB1*^*+*^ cells coexpress *MX1* whereas all *MX1*^*+*^ cells are *CPEB1*^*+*^. (**C’**) dFISH of *MX1* (magenta) and *surfactant b* (green). A subset of *surfactant b*^*+*^ cells coexpresses *MX1* whereas virtually all *MX1*^*+*^ cells are *surfactant b*^*+*^. (**A’–C’**) Side panels are high-magnification views of outlined areas. (**A’–C’**) Nuclei are counterstained with DAPI (gray). Scale bars, 200 μm (**A–C**), 50 μm for overview images, 20 μm for side panels (**A’–C’**). Underlying data can be found in S1 Data. dFISH, double FISH.
(PDF)

S7 Fig Vitellaria develop postembryonically and produce differentiating yolk cells during sexual maturation. (**A**, **B**) Maximum intensity projections of confocal sections showing dFISH of *klf4l* (green) with *nanos*, or vitellaria markers *CPEB1*, *surfactant b*, or *MX1* (magenta) in the ventral posterior region of hatchlings (**A**) or juveniles (**B**). Dashed line denotes planarian boundary. (**A**) Hatchlings do not express any of the vitellaria markers tested and are devoid of vitellaria. (**B**) *klf4l*^*+*^/*nanos*^*+*^ yolk cell progenitors, as well as *klf4l*^–^/*nanos*^*+*^, *CPEB1*^*+*^, and *surfactant b*^*+*^ differentiating yolk cells are detected in all juveniles. Only a fraction of juveniles express *MX1*^*+*^ yolk cells (**B**). (**A**, **B**) Nuclei are counterstained with DAPI (gray). Scale bars, 100 μm. dFISH, double FISH; *klf4l*, *klf4-like*.
(PDF)

S8 Fig RNAi triggers targeting nonoverlapping *klf4l* sequences lead to similar defects in vitellogenesis. Maximum intensity projections of confocal sections showing FISH of *MX1* (green; mature yolk cells) in ventral tail region of control and *klf4l* RNAi animals. RNAi triggered by dsRNA targeting near-full-length *klf4l*, an amino-terminal portion of *klf4l*, or a carboxyl-terminal portion of *klf4l* leads to similar loss of *MX1*^*+*^ yolk cells. Dashed line denotes planarian boundary. *N* = 2 experiments, *n* = 6 to 7 planarians. (**A**, **B**) Nuclei are counterstained with DAPI (gray). Scale bars, 200 μm. FISH, fluorescent RNA in situ hybridization; *klf4l*, *klf4-like*; RNAi, RNA interference.
(PDF)

S9 Fig Yolk cells express neoblast/germ cell markers. (**A**) Single confocal sections showing dFISH of neoblast and germ cell marker *gH4* (magenta) and *klf4l* (green). *gH4* is expressed at high levels in neoblasts as well as in spermatogonia and oogonia. *klf4l*^*+*^ cells in the testes (top panels), ovarian field, and ovary (ov) (bottom panel) coexpress *gH4* (yellow arrowheads). Note the absence of *gH4* in differentiated somatic cells found in the brain (br) and tuba (tu). Nuclei are counterstained with DAPI (gray). (B, C) Maximum intensity projections of confocal sections showing dFISH of *klf4l* and neoblast/germline markers *piwi-1* or *gH4* in the vitellaria. (**D**) Maximum intensity projection of confocal sections showing dFISH of *gH4* (magenta) and *surfactant b* (green). Dashed line denotes planarian boundary. Scale bars, 50 μm (**A**), 200 μm (**B–D**). dFISH, double FISH; *klf4l*, *klf4-like*.
(PDF)

S10 Fig The vitellaria and ovary are comprised of 2 populations of *ophis*-expressing cells: *ophis*^*high*^ versus *ophis*^*low*^ cells. (**A–F**) Maximum intensity projections of confocal sections showing dFISH of vitellaria markers in the ventral posterior region of sexually mature planarians. Dashed line denotes planarian boundary. (**G**) Confocal section of an ovary depicting *LamA* expression (magenta) in somatic gonadal cells and *klf4l* expression (green) in early germ cells. (**H**) Confocal section of an ovary depicting *ophis*^*high*^ expression (magenta/gray) in somatic gonadal cell nuclei (filled arrowhead) and *ophis*^*low*^ expression in oogonia and oocytes (unfilled arrowhead). (**G**, **H**) Dashed line denotes ovary (white) and tuba (yellow) boundary. Nuclei are counterstained with DAPI (gray). Scale bars, 200 μm (**A–F**), 50 μm (**G, H**). dFISH, double FISH; *klf4l*, *klf4-like*.
(PDF)

S1 Data Quantifications and cell counts. (XLSX)

S1 Table Information for transcripts mentioned in this paper. (XLSX)

S2 Table Quantitative PCR primers. (XLSX)

## Abbreviations

dFISH: double FISH
dsRNA: double-stranded RNA
FISH: fluorescent RNA in situ hybridization
GSC: germline stem cell
*klf4*: *Krüppel-like factor 4*
*klf4l*: *klf4-like*
PGC: primordial germ cell
pHH3: phospho-Histone H3
qPCR: quantitative PCR
RNAi: RNA interference
