# Supplementary figures and images for: A Krüppel-like factor is required for development and regeneration of germline and yolk cells from somatic stem cells in planarians
Source: PLoS Biol. 2022 Jul 15;20(7):e3001472. doi: 10.1371/journal.pbio.3001472 (PMC9286257; doi:10.1371/journal.pbio.3001472)

# S1 Fig.

**A**

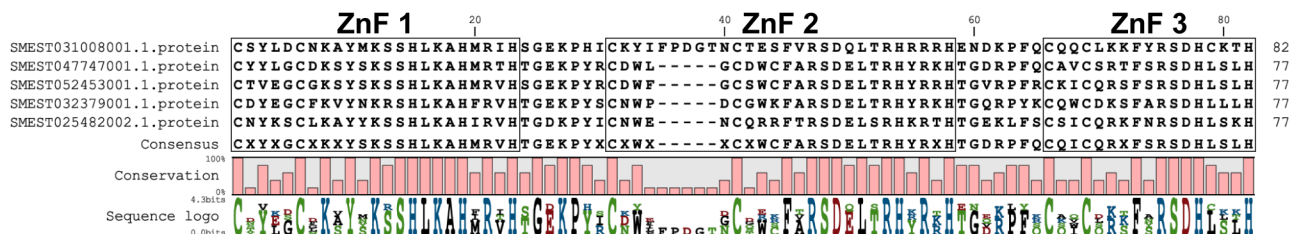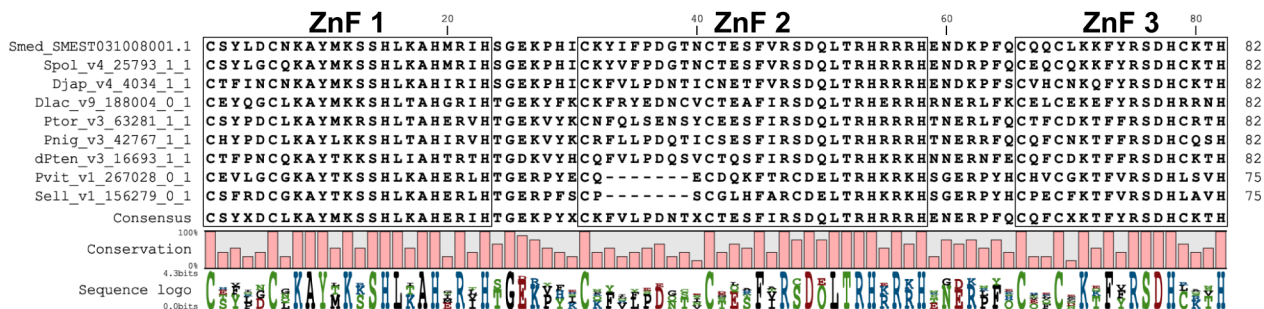

**B**

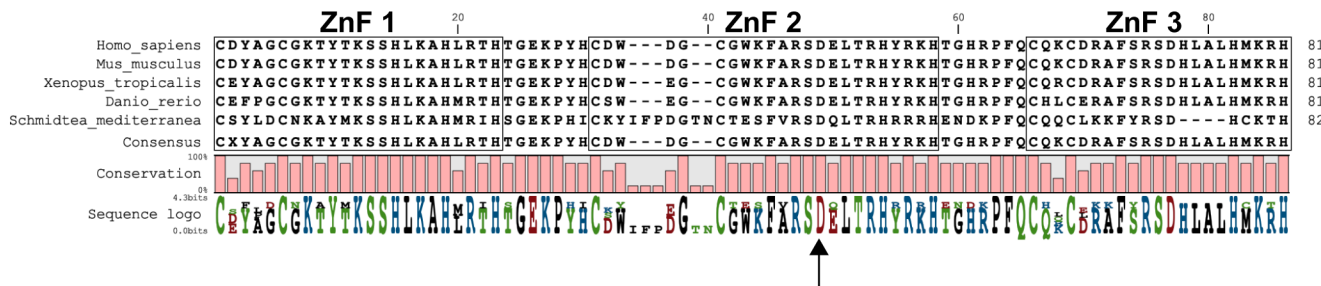

Supplement: S1 Fig — (A) The S. mediterranea genome contains 5 genes that encode Klf proteins [93]. Alignment of S. mediterranea Klf DBDs. The height of the red bars reflects percent conservation of the amino acid in the alignment. Sequence logo at bottom of the alignment depicts the consensus sequence. All Klfs are characterized by exactly 3 highly conserved C2H2 ZnF domains (each one outlined by a box) separated by intervening linker sequences and located at the carboxyl terminus. The black arrow points to a canonical aspartic acid residue (D) in ZnF2, which is important for Klf-DNA binding and is conserved in all filozoa (animals and their nearest unicellular relatives) [94]. Note the additional 5 amino acids in ZnF2 in S. mediterranea Klf4l (SMEST031008001.1.protein). These additional 5 residues in ZnF2 are a triclad innovation and are present in Klf4l homologs in the Platyhelminthes S. mediterranea (Smed), Schmidtea polychroa (Spol), Dugesia japonica (Djap), Dendrocoelum lacteum (Dlac), Planaria torva (Ptor), Polycelis nigra (Pnig), and Polycelis tenuis (Pten) (all from the Tricladida order), but not in Prostheceraeus vittatus (Pvit) and Stylochus ellipticus (Sell) (Polycladida). (B) The most significant human and mouse BLASTP hits for S. mediterranea Klf4l are Klf4 proteins. Alignment of Homo sapiens (NP_004226.3), Mus musculus (NP_034767.2), Xenopus tropicalis (NP_001017280.1), and Danio rerio (NP_571798.1) Klf4 DBDs with S. mediterranea (SMEST031008001.1) Klf4l DBD. DBD, DNA-binding domain; klf4l, klf4-like; ZnF, zinc finger. (PDF) [file pbio.3001472.s001.pdf]

S2 Fig.

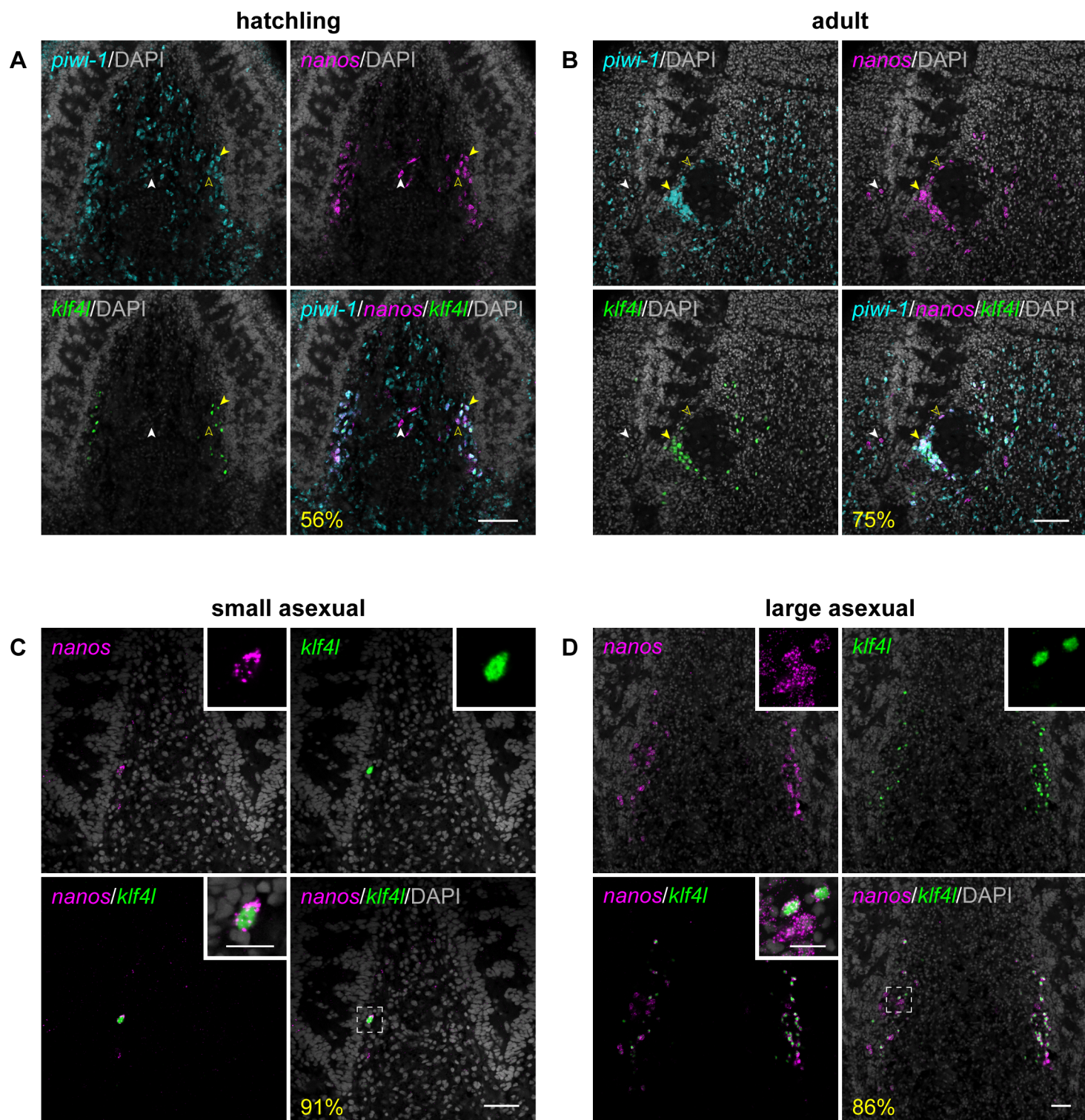

Supplement: S2 Fig — (A, B) Confocal section showing triple FISH of piwi-1 (cyan), klf4l (green), and nanos (magenta) in female germ cells in hatchlings and sexually mature ovary. klf4l is expressed in a subset of nanos+/piwi-1+ female germ cells (compare filled (klf4l+) to unfilled (klf4l–) yellow arrowhead). All klf4l+/nanos+ germ cells are piwi-1+. A small fraction of klf4l–/nanos+ cells do not express piwi-1 and are not germ cells (white arrowhead). (C, D) Confocal sections showing dFISH of klf4l (green) and nanos (magenta) in female germ cells (located mediolaterally along the planarian brain) in small (C) and large (D) asexual planarians. klf4l is expressed in a subset of nanos+ female germ cells. Insets show high-magnification views of heterogeneity of klf4l expression in nanos+ cells. (A–D) Percentages reflect nanos+ germ cells that are also klf4l+. Nuclei are counterstained with DAPI (gray). Scale bars, 100 μm (A, B), 50 μm for whole-brain images, 10 μm for insets (C, D). Underlying data can be found in S1 Data. FISH, fluorescent RNA in situ hybridization; klf4l, klf4-like. (PDF) [file pbio.3001472.s002.pdf]

S3 Fig.

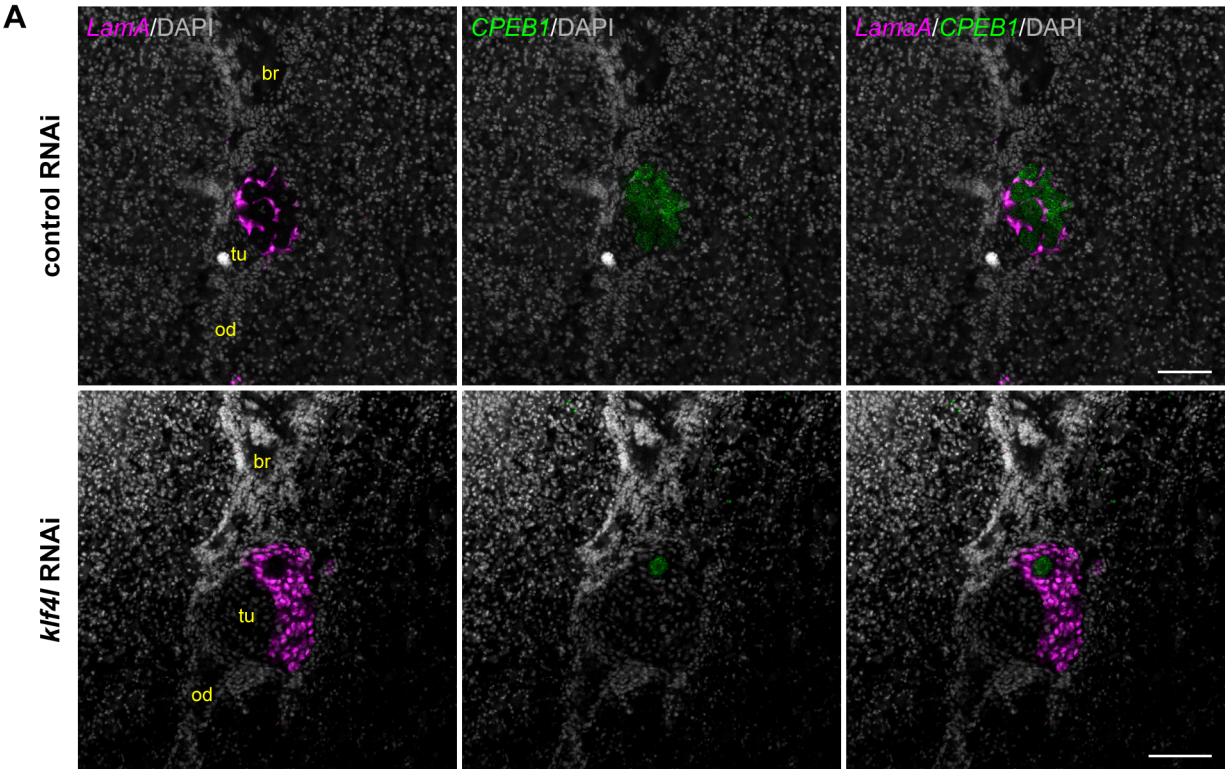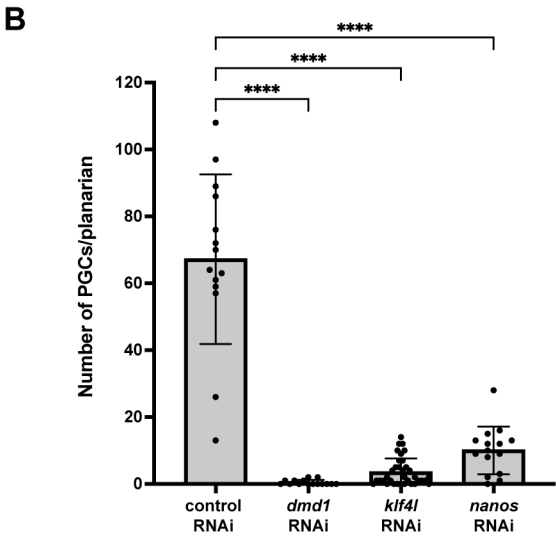

Supplement: S3 Fig — (A) Single confocal section of an ovary located at the posterior of the brain (br) and anterior to the tuba/oviduct (tu/od) showing dFISH of LamA (magenta; somatic gonadal cells) and CPEB1 (green; oocytes) in control and klf4l RNAi planarians. klf4l RNAi leads to oocyte loss and a nonautonomous increase in somatic support cells. Nuclei are counterstained with DAPI (gray). Scale bars, 100 μm. (B) Quantification of newly specified PGCs in head regenerates. Data are presented as mean ± SD. N = 3 to 5 experiments, n = 14 to 35 planarians. p < 0.0001, Welch ANOVA test. Underlying data can be found in S1 Data. dFISH, double FISH; klf4l, klf4-like; RNAi, RNA interference. (PDF) [file pbio.3001472.s003.pdf]

S4 Fig.

***klf4* RNAi (N-term):**

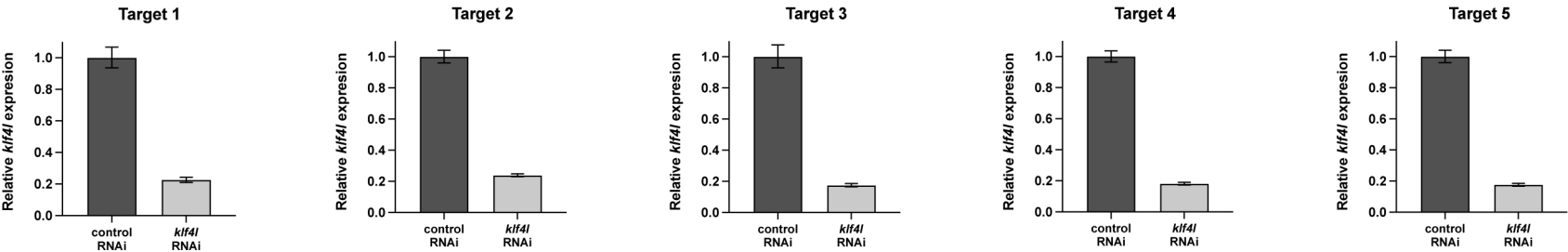

***klf4* RNAi (C-term):**

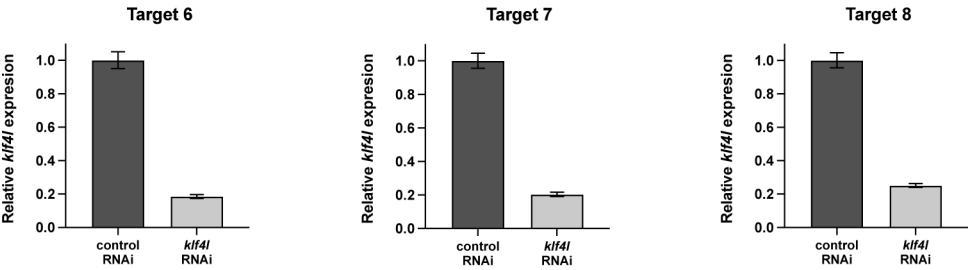

Supplement: S4 Fig — qPCR analysis of klf4l mRNA expression (normalized to β-tubulin) in control and klf4l RNAi animals depicting efficient knockdown of klf4l after RNAi treatment. Top: dsRNA targeting the amino terminus of klf4l was used for RNAi-mediated knockdown of klf4l, and qPCR primers targeting the carboxyl terminus were used to quantify klf4l expression levels. Bottom: dsRNA targeting the carboxyl terminus of klf4l was used for RNAi and qPCR primers targeting the amino terminus were used to quantify klf4l expression levels. N = 3 biological replicates (3 technical replicates each). Bar graphs depict 2−ΔΔCt values (normalized to control RNAi) with 95% confidence intervals. Underlying data can be found in S1 Data. klf4l, klf4-like; qPCR, quantitative PCR; RNAi, RNA interference. (PDF) [file pbio.3001472.s004.pdf]

S5 Fig.

**A**

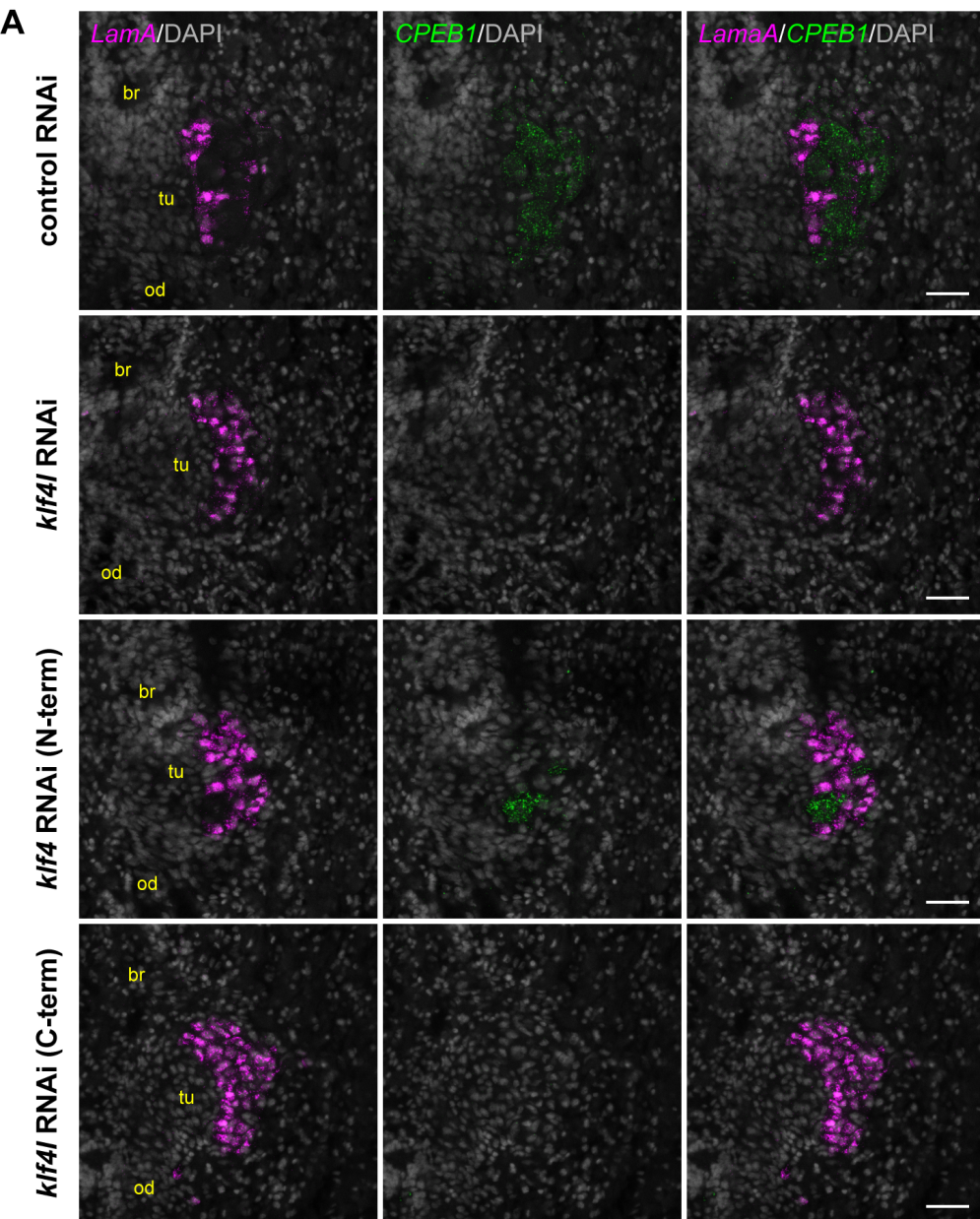

**B**

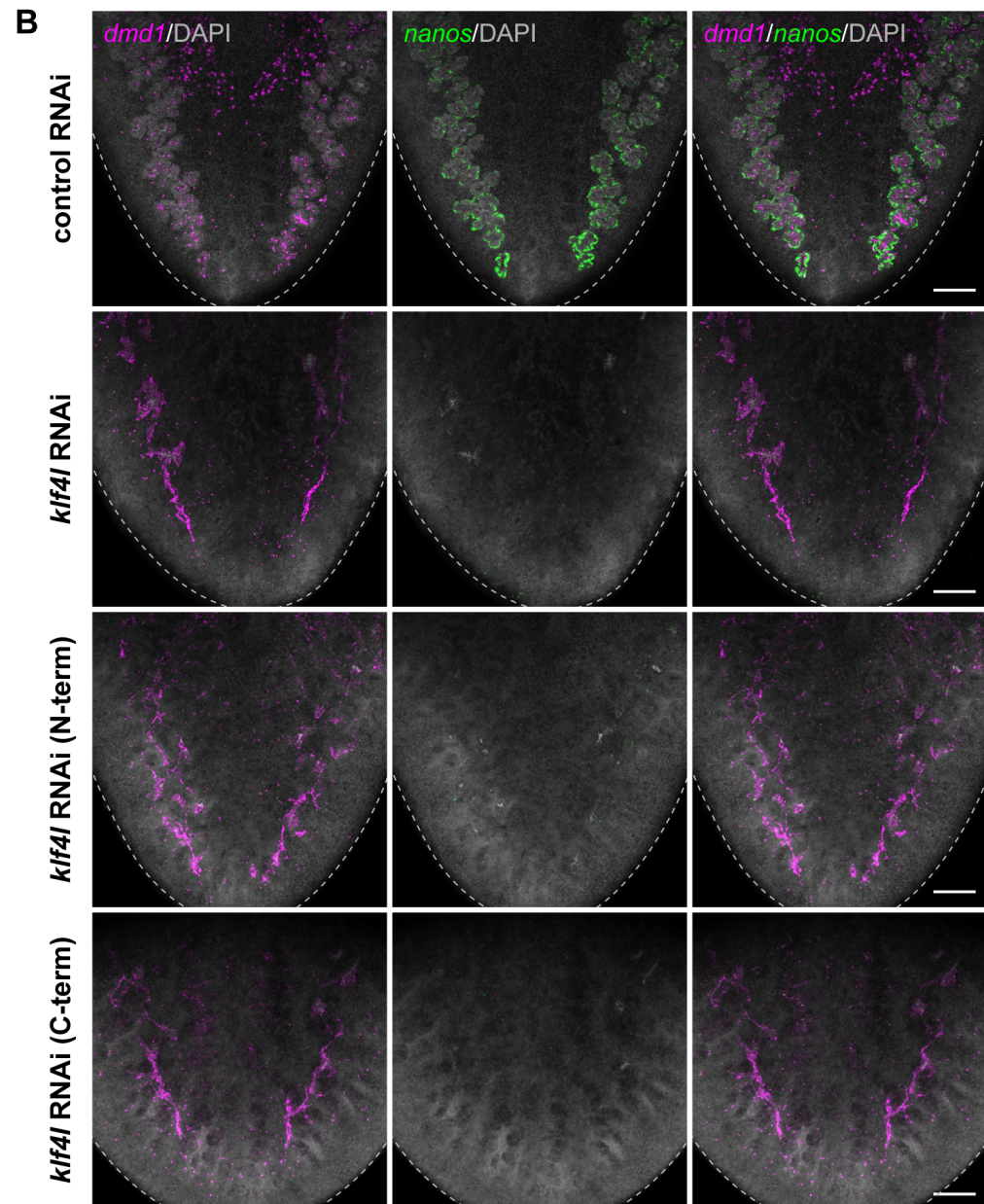

Supplement: S5 Fig — (A) Single confocal section of an ovary located posterior to the brain (br) and anterior to the tuba/oviduct (tu/od) showing dFISH of LamA (magenta; somatic gonadal cells) and CPEB1 (green; oocytes) in control and klf4l RNAi planarians. RNAi resulting from dsRNA targeting near-full-length klf4l, an amino-terminal portion of klf4l, or a carboxyl-terminal portion of klf4l all lead to similar defects in oogenesis. N = 2 experiments, n = 11–16 planarians. (B) Maximum intensity projections of confocal sections showing dFISH of dmd1 (magenta; somatic gonadal cells) and nanos (green) in a dorsal tail region where testes reside. All klf4l knockdowns lead to similar defects in spermatogenesis and “collapsed” testes due to male germ cell loss. Dashed line denotes planarian boundary. N = 2 experiments, n = 10 to 14 planarians. (A, B) Nuclei are counterstained with DAPI (gray). Scale bars, 50 μm (A), 200 μm (B). dFISH, double FISH; klf4l, klf4-like; RNAi, RNA interference. (PDF) [file pbio.3001472.s005.pdf]

S6 Fig.

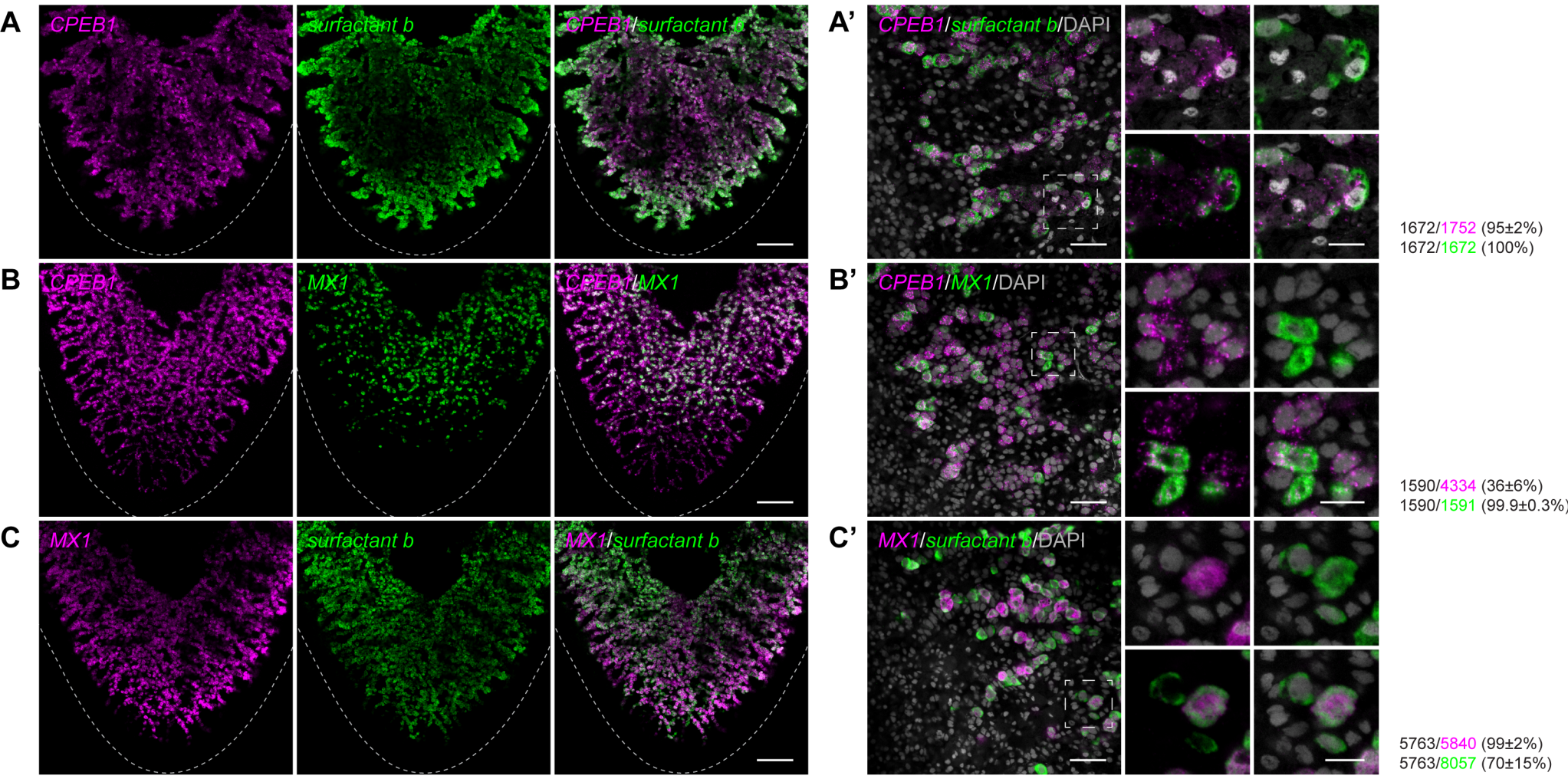

Supplement: S6 Fig — (A–C) Maximum intensity projections of confocal sections showing dFISH of vitellaria markers CPEB1 (A-B), surfactant b (A, C), and MX1 (B, C) in the ventral posterior region of sexually mature planarians. Dashed line denotes planarian boundary. (A’–C’) Single confocal sections of dFISH corresponding to A-C. (A’) dFISH of ventrally expressed CPEB1 (magenta) and surfactant b (green). Almost all CPEB1+ cells coexpress surfactant b and all surfactant b+ cells are CPEB1+. (B’) dFISH of CPEB1 (magenta) and MX1 (green). A subset of CPEB1+ cells coexpress MX1 whereas all MX1+ cells are CPEB1+. (C’) dFISH of MX1 (magenta) and surfactant b (green). A subset of surfactant b+ cells coexpresses MX1 whereas virtually all MX1+ cells are surfactant b+. (A’–C’) Side panels are high-magnification views of outlined areas. (A’–C’) Nuclei are counterstained with DAPI (gray). Scale bars, 200 μm (A–C), 50 μm for overview images, 20 μm for side panels (A’–C’). Underlying data can be found in S1 Data. dFISH, double FISH. (PDF) [file pbio.3001472.s006.pdf]

S7 Fig.

A. Hatchling

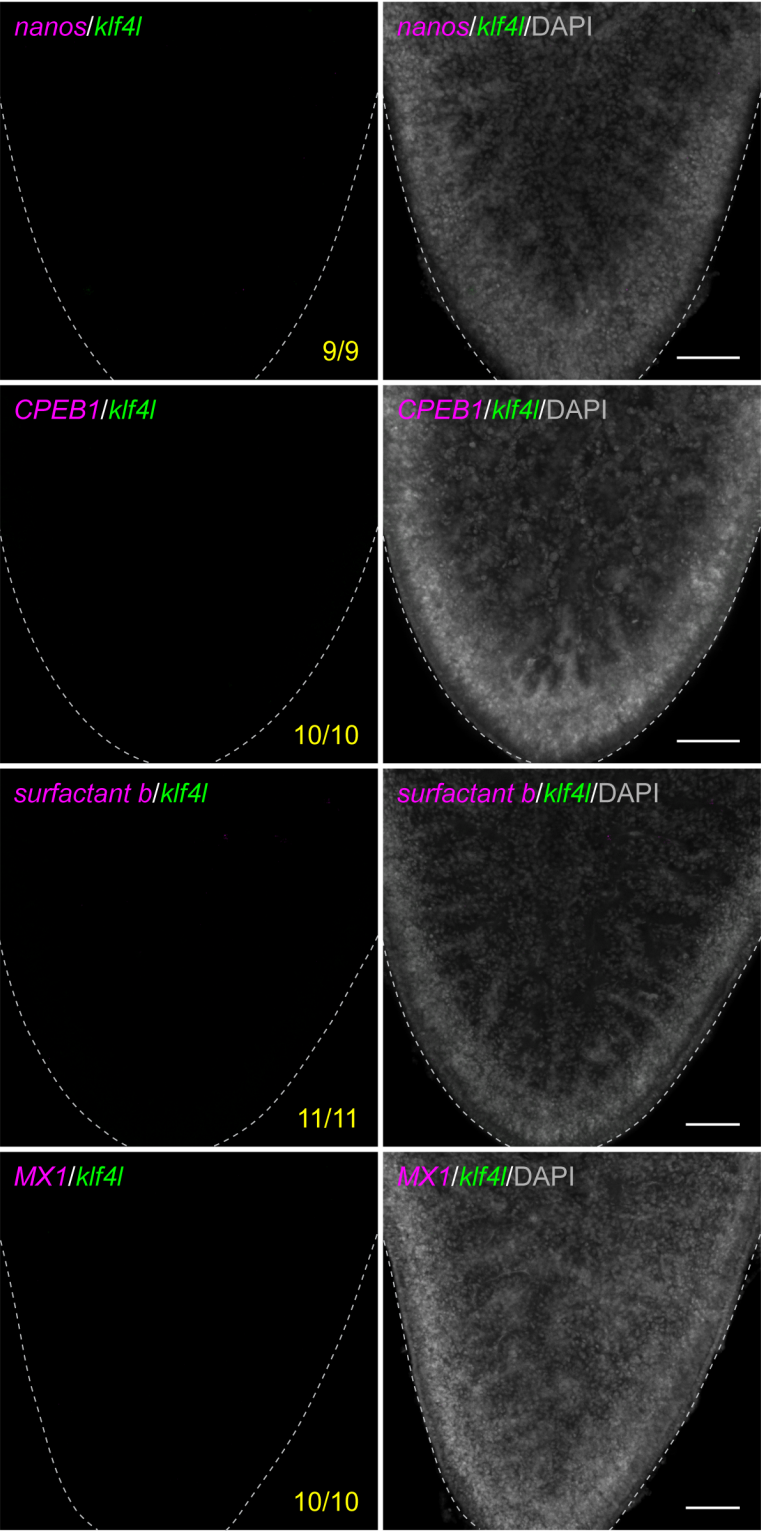

B. Juvenile

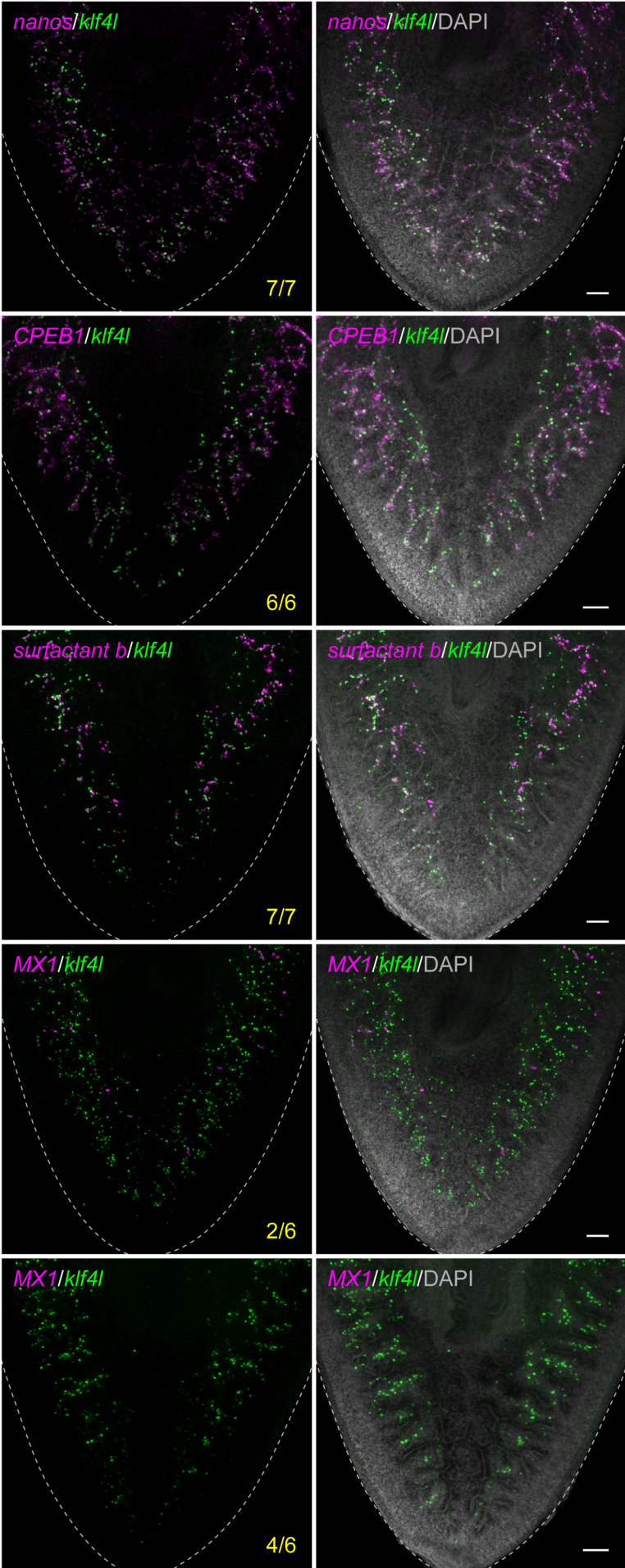

Supplement: S7 Fig — (A, B) Maximum intensity projections of confocal sections showing dFISH of klf4l (green) with nanos, or vitellaria markers CPEB1, surfactant b, or MX1 (magenta) in the ventral posterior region of hatchlings (A) or juveniles (B). Dashed line denotes planarian boundary. (A) Hatchlings do not express any of the vitellaria markers tested and are devoid of vitellaria. (B) klf4l+/nanos+ yolk cell progenitors, as well as klf4l–/nanos+, CPEB1+, and surfactant b+ differentiating yolk cells are detected in all juveniles. Only a fraction of juveniles express MX1+ yolk cells (B). (A, B) Nuclei are counterstained with DAPI (gray). Scale bars, 100 μm. dFISH, double FISH; klf4l, klf4-like. (PDF) [file pbio.3001472.s007.pdf]

S8 Fig.

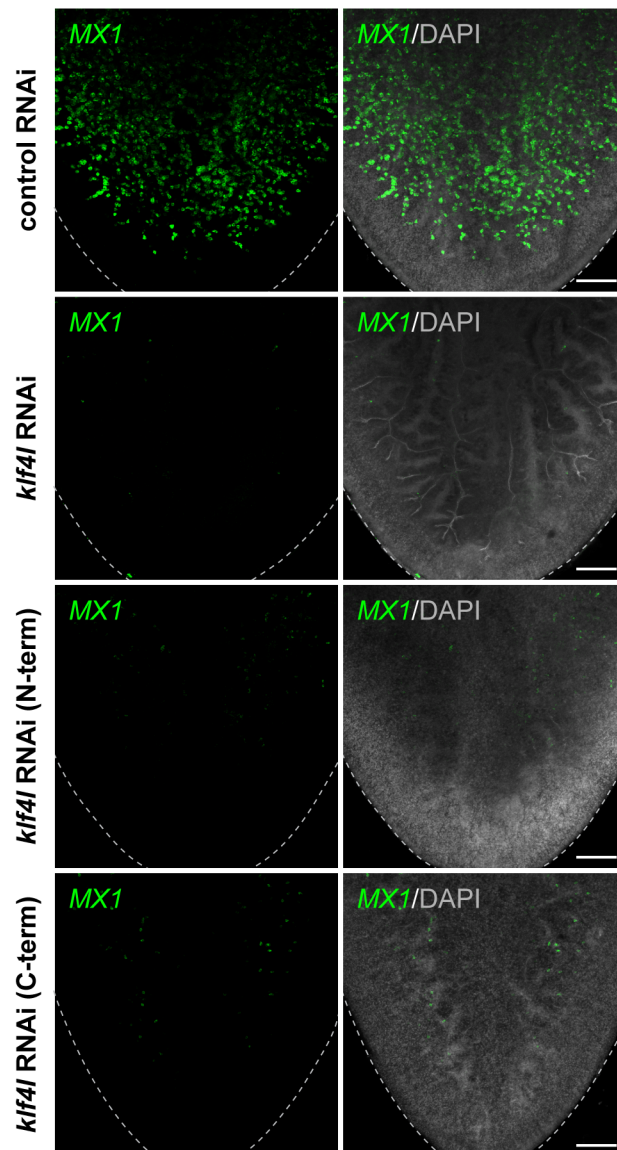

Supplement: S8 Fig — Maximum intensity projections of confocal sections showing FISH of MX1 (green; mature yolk cells) in ventral tail region of control and klf4l RNAi animals. RNAi triggered by dsRNA targeting near-full-length klf4l, an amino-terminal portion of klf4l, or a carboxyl-terminal portion of klf4l leads to similar loss of MX1+ yolk cells. Dashed line denotes planarian boundary. N = 2 experiments, n = 6 to 7 planarians. (A, B) Nuclei are counterstained with DAPI (gray). Scale bars, 200 μm. FISH, fluorescent RNA in situ hybridization; klf4l, klf4-like; RNAi, RNA interference. (PDF) [file pbio.3001472.s008.pdf]

S9 Fig.

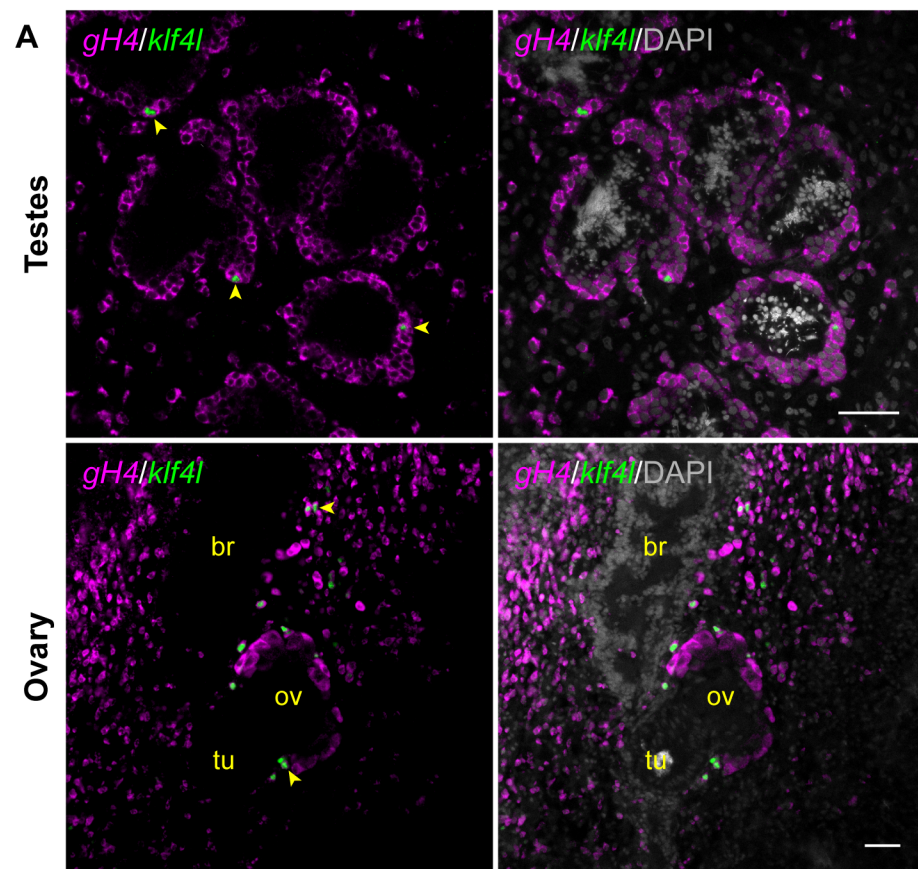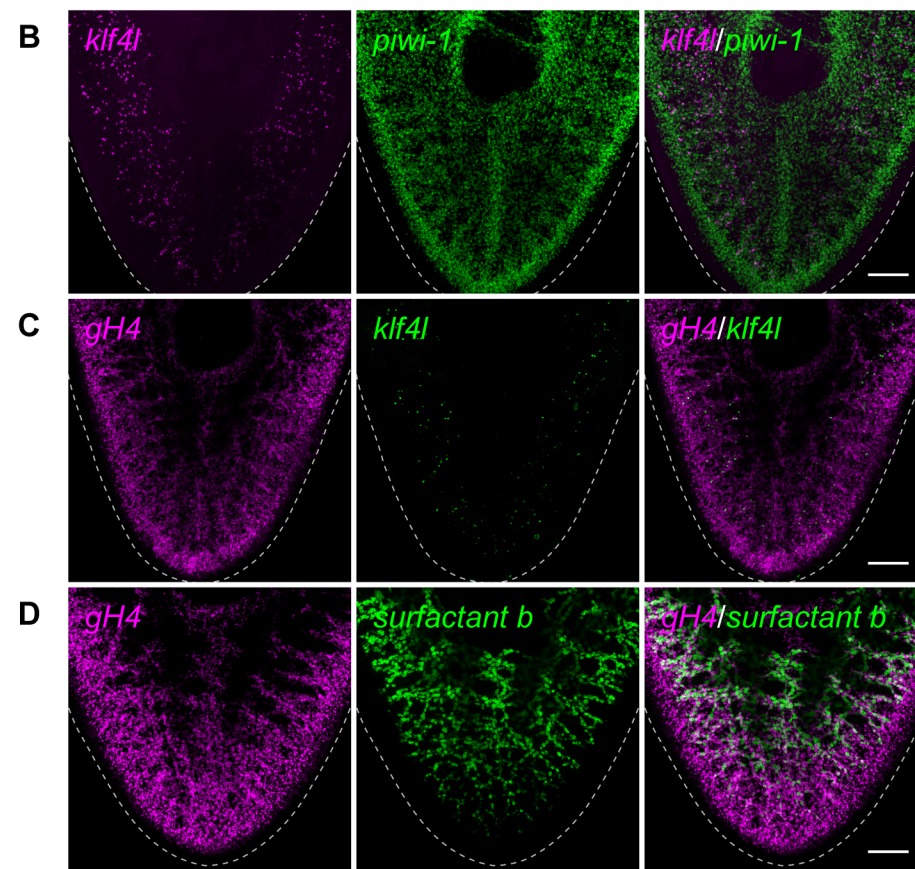

Supplement: S9 Fig — (A) Single confocal sections showing dFISH of neoblast and germ cell marker gH4 (magenta) and klf4l (green). gH4 is expressed at high levels in neoblasts as well as in spermatogonia and oogonia. klf4l+ cells in the testes (top panels), ovarian field, and ovary (ov) (bottom panel) coexpress gH4 (yellow arrowheads). Note the absence of gH4 in differentiated somatic cells found in the brain (br) and tuba (tu). Nuclei are counterstained with DAPI (gray). (B, C) Maximum intensity projections of confocal sections showing dFISH of klf4l and neoblast/germline markers piwi-1 or gH4 in the vitellaria. (D) Maximum intensity projection of confocal sections showing dFISH of gH4 (magenta) and surfactant b (green). Dashed line denotes planarian boundary. Scale bars, 50 μm (A), 200 μm (B–D). dFISH, double FISH; klf4l, klf4-like. (PDF) [file pbio.3001472.s009.pdf]

S10 Fig.

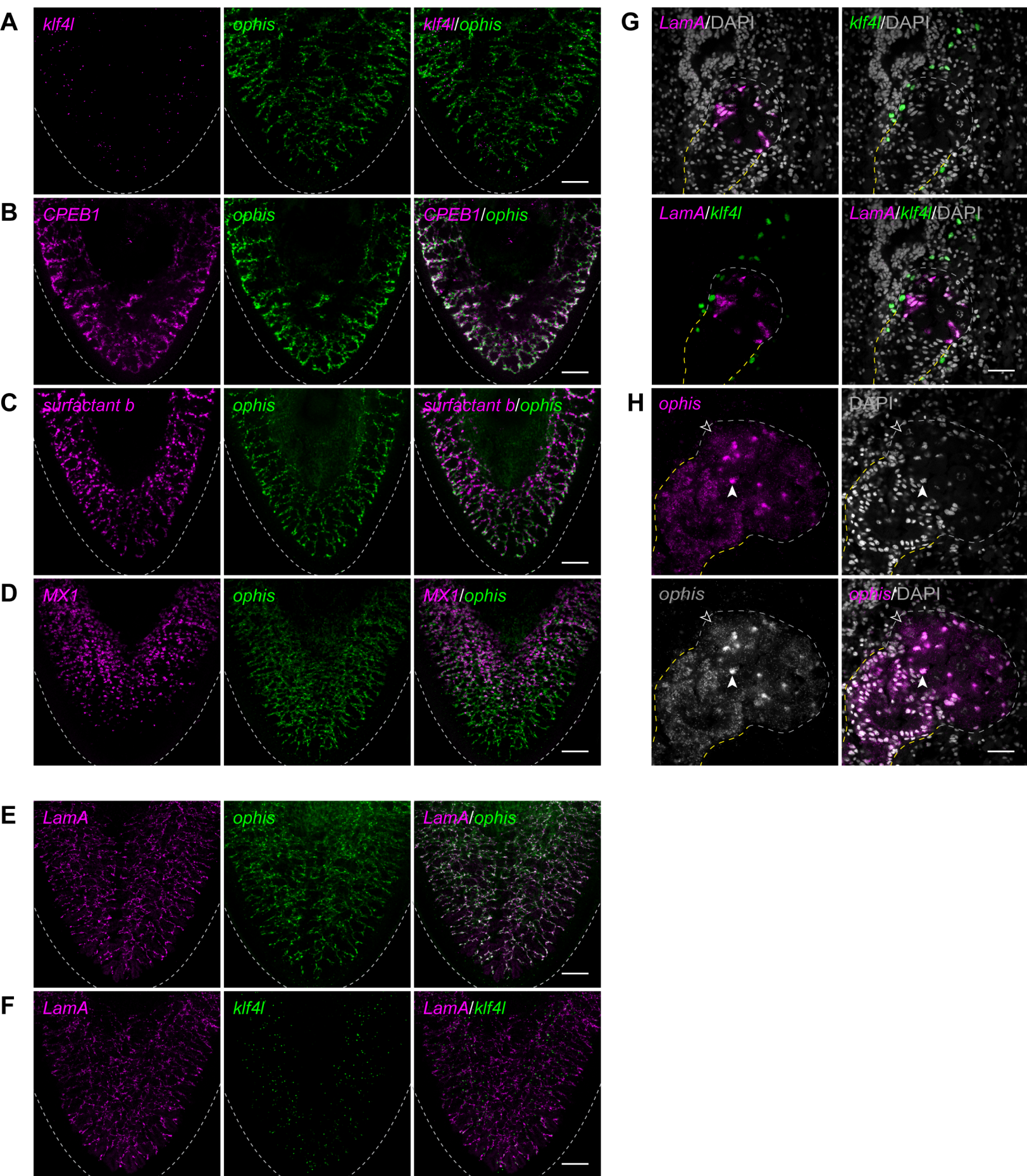

Supplement: S10 Fig — (A–F) Maximum intensity projections of confocal sections showing dFISH of vitellaria markers in the ventral posterior region of sexually mature planarians. Dashed line denotes planarian boundary. (G) Confocal section of an ovary depicting LamA expression (magenta) in somatic gonadal cells and klf4l expression (green) in early germ cells. (H) Confocal section of an ovary depicting ophishigh expression (magenta/gray) in somatic gonadal cell nuclei (filled arrowhead) and ophislow expression in oogonia and oocytes (unfilled arrowhead). (G, H) Dashed line denotes ovary (white) and tuba (yellow) boundary. Nuclei are counterstained with DAPI (gray). Scale bars, 200 μm (A–F), 50 μm (G, H). dFISH, double FISH; klf4l, klf4-like. (PDF) [file pbio.3001472.s010.pdf]
